# A Markov chain for numerical chromosomal instability in clonally expanding populations

**DOI:** 10.1371/journal.pcbi.1006447

**Published:** 2018-09-11

**Authors:** Sergi Elizalde, Ashley M. Laughney, Samuel F. Bakhoum

**Affiliations:** 1 Department of Mathematics, Dartmouth College, Hanover, New Hampshire, United States of America; 2 Cancer Biology and Genetics, Memorial Sloan Kettering Cancer Center, New York, New York, United States of America; 3 Human Oncology and Pathogenesis Program, Memorial Sloan Kettering Cancer Center, New York, New York, United States of America; 4 Department of Radiation Oncology, Memorial Sloan Kettering Cancer Center, New York, New York, United States of America; Ottawa University, CANADA

## Abstract

Cancer cells frequently undergo chromosome missegregation events during mitosis, whereby the copies of a given chromosome are not distributed evenly among the two daughter cells, thus creating cells with heterogeneous karyotypes. A stochastic model tracing cellular karyotypes derived from clonal populations over hundreds of generations was recently developed and experimentally validated, and it was capable of predicting favorable karyotypes frequently observed in cancer. Here, we construct and study a Markov chain that precisely describes karyotypic evolution during clonally expanding cancer cell populations. The Markov chain allows us to directly predict the distribution of karyotypes and the expected size of the tumor after many cell divisions without resorting to computationally expensive simulations. We determine the limiting karyotype distribution of an evolving tumor population, and quantify its dependency on several key parameters including the initial karyotype of the founder cell, the rate of whole chromosome missegregation, and chromosome-specific cell viability. Using this model, we confirm the existence of an optimal rate of chromosome missegregation probabilities that maximizes karyotypic heterogeneity, while minimizing the occurrence of nullisomy. Interestingly, karyotypic heterogeneity is significantly more dependent on chromosome missegregation probabilities rather than the number of cell divisions, so that maximal heterogeneity can be reached rapidly (within a few hundred generations of cell division) at chromosome missegregation rates commonly observed in cancer cell lines. Conversely, at low missegregation rates, heterogeneity is constrained even after thousands of cell division events. This leads us to conclude that chromosome copy number heterogeneity is primarily constrained by chromosome missegregation rates and the risk for nullisomy and less so by the age of the tumor. This model enables direct integration of karyotype information into existing models of tumor evolution based on somatic mutations.

## Author summary

Chromosomal instability (CIN) is a hallmark of cancer and it results from persistent chromosome segregation errors during cell division. CIN has been shown to play a key role in drug resistance and tumor metastasis. While our understanding of CIN on the cellular level has grown over the past decade, our ability to predict the behavior of tumors containing billions of cells remains limited due to the paucity of adequate mathematical models. Here, we develop a Markov-chain model that is capable of providing exact solutions for long-term chromosome copy number distributions during tumor growth. Using this model we confirm the presence of optimal chromosome missegregation rates that balance genomic heterogeneity required for tumor evolution and survival. Interestingly, we show that chromosome copy number heterogeneity is primarily influenced by the rate of chromosome segregation errors rather than the age of the tumor. At chromosome missegregation rates frequently observed in cancer, tumors can acquire maximal genomic heterogeneity after a few hundred cell divisions. This model enables the integration of selection imparted by CIN into existing models of tumor evolution based on somatic mutations to explore their mutual effects.

## Introduction

Cancer genomic heterogeneity, which is often driven by genomic instability, enables Darwinian selection, leading to tumor metastasis and increased resistance to therapeutic pressures [1–3]. A frequent, yet understudied source of genetic heterogeneity is numerical chromosomal instability, which allows cancer cells to rapidly vary the number of copies of each chromosome (karyotype) through whole chromosome missegregation events during mitosis [4–7]. This karyotypic heterogeneity can lead to tumor cells with varying fitness levels depending on the potency and distribution of oncogenes (proliferative) and tumor suppressor genes (anti-proliferative) on individual chromosomes [8]. Despite its importance, the contribution of numerical chromosomal instability toward tumor evolution has been poorly understood due to limitations in experimental and theoretical models that attempt to understand this process on the systems level.

Chromosome missegregation was first incorporated into a model of tumor evolution by Gusev *et al*. [9] and later in a continuous time model by Desper *et al*. [10]. While helpful, these models neglected the observed phenomenon that having more copies of chromosomes encoding a higher fraction of oncogenes is advantageous for the cell, while having more copies of chromosomes encoding tumor suppressor genes increases its chances of dying [8]. Laughney et al. addressed this limitation by building a stochastic model that tracks single cell karyotypes derived from clonal populations over hundreds of generations, while simultaneously allowing the cumulative proliferative or anti-proliferative effects of genes encoded on individual chromosomes to alter cellular viability [4]. This model incorporates chromosome-specific scores derived from a recent genomic analysis by Davoli *et al*. [8], which weighs individual chromosomes based on the potency and chromosomal distribution of oncogenes (proliferative, contributing positively) and tumor suppressor genes (anti-proliferative, contributing negatively). The scores of the individual chromosomes are then aggregated to determine the survival probability of each cell. In its most basic form, the model assumes the following:

1. When a cell divides and gives rise to two daughter cells, each individual chromosome copy has a fixed probability of undergoing a missegregation event. Such an event leads to disproportionate inheritance, causing the two daughter cells to end up with one too many or one too few copies of the missegregated chromosome.
2. Cells are considered nonviable if they completely lose any given chromosome (a process known as nullisomy), as they would be missing a number of essential genes, or if they have more than 8 copies of any given chromosome. Sensitivity analysis for these assumptions has been performed for key conclusions [4].

The model by Laughney *et al*. unveiled several key observations which were validated experimentally. First, it revealed a highly favorable, and commonly observed near-triploid state, onto which evolving cells converge. This is in line with enrichments for near-triploid karyotypes observed in human tumors deposited in the Mitelman database, as well as tumor ploidy inferred from bulk DNA sequencing of TCGA tumors [11, 12]. It also predicted the existence of an optimal missegregation rate —which maximizes cell viability with the generation of heterogeneity—that agreed with the experimentally measured chromosome missegregation rates observed in human cancer-derived cell lines [13, 14]. Finally, it was directly validated by predicting the frequency at which single cells deviate from the modal chromosome numbers for any given chromosome in an expanding clonal population after 25 cell divisions, as experimentally measured in single-cell-derived clones by fluorescence *in situ* hybridization. This model, however, was unable to predict the limiting distribution of cellular karyotypes in a tumor population or to complement models of tumor evolution based on somatic mutations, which occur with relatively low frequency, given the sheer number of cells that must be tracked for many generations in order to reach a probabilistic conclusion. It was also unable to test the dependence of large tumor cell populations on multiple parameters due to the sheer computational power required to perform such simulations.

In this paper, we construct and mathematically analyze a Markov chain that describes the evolution of the karyotype of a random cell in the above stochastic model. A special case of this Markov chain was briefly mentioned in [4] and used in some computations. However, no mathematical analysis was given, where the focus was to obtain a biological understanding of the role of numerical chromosomal instability in tumor evolutionary dynamics.

The structure of the paper is as follows: in the Methods section, we start by describing a simplified version of the model and its associated Markov chain without chromosome-specific influence on cellular viability. Then we describe the full model which enables chromosome-specific scores to alter cellular viability. In the Results section we analyze both models. First we show that the simplified Markov chain, after some slight adjustments, has interesting mathematical properties; for example, the limiting cellular karyotype does not depend on the chromosome missegregation rate. We study this limiting karyotype, as well as its dependence on the maximum allowed number of copies of each chromosome. Next we focus on the full model, showing that, interestingly, the limiting distribution of cellular karyotypes is no longer independent of missegregation rate in this scenario. We show that by varying key parameters of the model, namely the missegregation rate (or probability, *p*) and the chromosome scores, very different behaviors are obtained in the limit. In particular, for parameters observed in human cancer cells, the resulting limiting behaviors are more realistic than those predicted in [9]. Finally, using our model, we find that maximal karyotype heterogeneity can indeed be achieved after a small number of cell divisions at chromosome missegregation rates frequently observed in cancer. This suggests that chromosome missegregation is more consequential toward genomic heterogeneity than the tumor lifetime, as tumors with low missegregation rates cannot reach maximal heterogeneity even after tens of thousands of generations of cell division. The Discussion section explains these conclusions, and compares our model to others in the literature.

## Methods

### The basic model

Let us begin by describing a simplified version of the stochastic model, which is also used in [4].

The karyotype of a cell is the vector (*n*_1_, …, *n*_23_) where *n*_*k*_ is the number of copies of chromosome *k* that it contains. Starting from a founder cell with a given karyotype, at each generation, all the cells in the colony divide, giving rise to two cells. When a cell divides, each of the *n*_*k*_ copies of chromosome *k*, for 1 ≤ *k* ≤ 23, splits into two copies. In normal circumstances, each copy goes to one of the daughter cells, so the daughters have the same karyotype as the mother. However, with probability *p*, the two copies go to the same daughter cell, while the other daughter receives no copies. Such an event is called a *missegregation*, and *p* is called the *missegregation rate* (per chromosome copy per cell division). Note that at each cell division, each copy of each chromosome undergoes a missegregation with probability *p*, and these events are independent of each other. If the number of copies of a chromosome in a cell reaches 0 or goes above the maximum allowed number of copies, *N*, the cell automatically dies and no longer reproduces. Thus, for a cell to be viable, it must have 1 ≤ *n*_*k*_ ≤ *N* for all *k*.

The basic stochastic model described in this section does not include chromosome-specific scores; these will be included in the next section. In the basic model, the only way for a cell to die is if the number of copies of a chromosome leaves the range [1, *N*]. We construct a Markov chain M that models the proportion of copies of a given chromosome in the colony. The following simplifications will make our model more tractable:

1. Since, by hypothesis, missegregation events that take place for the different chromosomes are independent, we consider only one type of chromosome (say, chromosome *k*) at a time. Let us suppose, for now, that cells only have one type of chromosome, and so the only information that we need about the cell is whether it is dead, and otherwise how many copies of the chromosome it has. Thus, our Markov chain has an absorbing state labeled 0, corresponding to dead cells, and *N* non-absorbing states, with a label *i*, where 1 ≤ *i* ≤ *N*, that indicates the number of copies of the chromosome. This simplification allows us to work with only *N* non-absorbing states instead of *N*^23^. We will be able obtain the probability of a given karyotype (*n*_1_, …, *n*_23_), with 1 ≤ *n*_*k*_ ≤ *N* for all *k*, by multiplying the probability that the Markov chain corresponding to chromosome *k* is in state *n*_*k*_ for 1 ≤ *k* ≤ 23.
2. We follow a random branch in the evolution process by starting with the founder cell and randomly considering one of the two daughters at each division. The number of copies of chromosome *k* in a cell is affected only by the number of copies of that chromosome in the mother and by the missegregation rate. The Markov chain M at time *g* will give the probability that a random branch, after *g* generations, ends at cell with *i* copies of chromosome *k*, for each 1 ≤ *i* ≤ *N*, or at a dead cell with a disallowed number of copies of chromosome *k*.
3. To simplify the transition probabilities, we disregard the highly unlikely event that multiple copies of the same chromosome in a cell missegregate simultaneously. To that end, we disregard terms that are quadratic in *p*, which are negligible when *p* is very small.

With the above assumptions, the transition matrix **M** for the non-absorbing states has entries

$$M_{ij}=\{\begin{array}{cc} 1-ip & \text{if}\;i=j,\\ ip/2 & \text{if}\;|i-j|=1,\\ 0 & \text{if}\;|i-j|\geq 2, \end{array}$$

for 1 ≤ *i*, *j* ≤ *N*, where *M*_*ij*_ is the probability of transitioning from state *i* to state *j*. Adding an extra row and column corresponding to the absorbing state 0, we get the matrix

$${\text{M}}^{\prime }=[\begin{array}{cc} \begin{array}{c} 1 \end{array} & \begin{array}{c} 0\;\cdots \;0 \end{array}\\ \begin{array}{c} p/2\\ 0\\ \vdots \\ 0\\ Np/2 \end{array} & \begin{array}{c} \text{M} \end{array} \end{array}].$$

For example, if the maximum number of chromosomes is *N* = 8, which is the bound used in [4], we have

$$\begin{array}{c} M^{\prime }=\left[\begin{array}{ccccccccc} 1 & 0 & 0 & 0 & 0 & 0 & 0 & 0 & 0\\ p/2 & 1-p & p/2 & 0 & 0 & 0 & 0 & 0 & 0\\ 0 & p & 1-2p & p & 0 & 0 & 0 & 0 & 0\\ 0 & 0 & 3p/2 & 1-3p & 3p/2 & 0 & 0 & 0 & 0\\ 0 & 0 & 0 & 2p & 1-4p & 2p & 0 & 0 & 0\\ 0 & 0 & 0 & 0 & 5p/2 & 1-5p & 5p/2 & 0 & 0\\ 0 & 0 & 0 & 0 & 0 & 3p & 1-6p & 3p & 0\\ 0 & 0 & 0 & 0 & 0 & 0 & 7p/2 & 1-7p & 7p/2\\ 4p & 0 & 0 & 0 & 0 & 0 & 0 & 4p & 1-8p \end{array}\right]. \end{array}$$

Indeed, each copy of the chromosome in a cell will produce 0, 1 or 2 copies in a random daughter with probability *p*/2, 1 − *p* and *p*/2, respectively. If a cell has *i* copies of the chromosome, since each one of these copies missegregates independently, the probability that a random daughter has *j* copies is given by the coefficient of *x*^*j*^ in the polynomial $(\frac{p}{2}+\left(1-p\right)x+\frac{p}{2}x^{2}{)}^{i}$. Neglecting quadratic terms in *p*, we have

$$(\frac{p}{2}+\left(1-p\right)x+\frac{p}{2}x^{2}{)}^{i}\approx \frac{ip}{2}x^{i-1}+\left(1-ip\right)x^{i}+\frac{ip}{2}x^{i+1}.$$

This gives the rows of **M**′, except for the first row, which is trivial because a dead cell does not divide, and the last row, which takes into account that a cell with *N* + 1 copies is considered dead.

To describe the evolution of the karyotypes of tumor cells with 23 types of chromosomes in this basic model, we consider the product of 23 Markov chains, each of them isomorphic to M. We can do this because missegregation events involving different chromosomes are independent, and the number of copies of each chromosome evolves according to M. Product states where at least one of the components corresponds to a dead (i.e. absorbing) state are regarded as dead states in the product chain. Thus, even though the Markov chain for chromosome *k* does not capture the fact that a cell may die because of a disallowed number of copies of another chromosome, this event is taken into account in the product of the 23 chains. One way to think about it is by pretending that cells with no copies of a chromosome still divide as usual, but they give rise to two dead cells with no copies of that chromosome.

### The model with chromosome scores

In the basic model from the previous section, the only way for a cell to die is if the number of copies of a chromosome reaches 0 or goes above *N*. A more realistic model should include the possibility that a cell dies for other reasons. In fact, the karyotype of the cell is postulated to have an influence on its survival probability. It has been proposed [8] that having more copies of certain oncogenic chromosomes is subject to positive selection as evidenced by a pan-cancer analysis of chromosome-level amplifications, whereas having more copies of other tumor-suppressive chromosomes is subject to negative selection.

In this section we construct a more general Markov chain which takes these factors into account. This Markov chain describes the evolution of the number of chromosome copies in random cells in the stochastic model of Laughney *et al*. [4]. As in that model, we assign a score *s*_*k*_ to each chromosome *k*, which is positive for oncogenic chromosomes and negative for tumor-suppressive ones, so that the total score of a cell with karyotype (*n*_1_, …, *n*_23_) is $S=\sum_{k=1}^{23}s_{k}n_{k}$. Numerical values of *s*_*k*_ were experimentally inferred by Davoli *et al*. [8]. Here we describe the Markov chain in a more abstract setting where the *s*_*k*_ are left as parameters.

The survival probability of the cell with score *S* at a given generation is *Q*_surv_ = *e*^*c*+*dS*^ for some constants *c* < 0 and *d* > 0, which again are parameters of the model. With probability 1 − *Q*_surv_, the cell spontaneously dies at that generation. With probability *Q*_surv_, the cell divides as usual, with missegregation events taking place as in the model without scores. Note that it is still possible for the daughter cells to die if the number of copies of a chromosome leaves the range [1, *N*], but this cause of death is unrelated to the survival probability *Q*_surv_.

A key obervation that will make the size of our Markov chains tractable is that

$$Q_{\text{surv}}=e^{c+d\sum_{k=1}^{23}s_{k}n_{k}}=\prod_{k=1}^{23}e^{c_{k}+ds_{k}n_{k}}=\prod_{k=1}^{23}q_{k}\left(n_{k}\right),$$ (1)

where the *c*_*k*_ are arbitrary constants with *c*_1_ + ⋯ + *c*_23_ = *c*, and we write $q_{k}(i)=e^{c_{k}+ds_{k}i}$ to denote the contribution to the survival probability coming from chromosome *k*. It will be convenient to write *q*_*k*_(*i*) = *Cμ*^*i*^ for constants $C=e^{c_{k}}$ and $\mu =e^{ds_{k}}$ (note that *μ* > 1 if and only if chromosome *k* is oncogenic).

Eq (1) allows us to break up the model with chromosome scores into 23 independent Markov chains $A^{\left(k\right)}$, one for each chromosome type. In $A^{\left(k\right)}$, a cell in state *i* has probability *q*_*k*_(*i*) of dividing as usual (as in the Markov chain M from the basic model), and probability 1 − *q*_*k*_(*i*) of spontaneously dying, which is represented by a transition to the absorbing state 0. The evolution of karyotypes in the colony is then described by the product of the 23 Markov chains $A^{\left(k\right)}$ for 1 ≤ *k* ≤ 23. Again, a product state where at least one of the coordinates corresponds to the absorbing state of some $A^{\left(k\right)}$ is regarded as a dead state in the product chain. With this setup, a cell with karyotype (*n*_1_, …, *n*_23_) has probability *Q*_surv_ = ∏_*k*_ *q*_*k*_(*n*_*k*_) of surviving and dividing as in the model without scores, with each chromosome type behaving independently, and probability 1 − *Q*_surv_ of spontaneously dying. Since viable states in the product chain correspond to products of viable states in the chains $A^{\left(k\right)}$, the proportion of cells with a given karyotype (*n*_1_, …, *n*_23_) after *g* generations (as a fraction of 2^*g*^) is given by the product for 1 ≤ *k* ≤ 23 of the probability that the Markov chain $A^{\left(k\right)}$ is in state *n*_*k*_. This means that the simplification 1 described in the previous section is still applicable in the model with chromosome scores.

When it creates no confusion, we will simply write A instead of $A^{\left(k\right)}$. The transition matrix of this Markov chain restricted to the non-absorbing states is **A**, with entries defined as

$$A_{ij}=\{\begin{array}{cc} \left(1-ip\right)\;q_{k}\left(i\right) & \text{if}\;i=j,\\ ip\;q_{k}\left(i\right)/2 & \text{if}\;|i-j|=1,\\ 0 & \text{if}\;|i-j|\geq 2, \end{array}$$

for 1 ≤ *i*, *j* ≤ *N*. We can express **A** as **A** = **DM**, where **D** is the diagonal matrix with *D*_*ii*_ = *q*_*k*_(*i*) for 1 ≤ *i* ≤ *N*, and **M** is the matrix from the basic model.

If the value of the parameter *c* is such that *Q*_surv_ ≤ 1 for all valid karyotypes, then it is possible to choose the constants *c*_*k*_ so that *q*_*k*_(*i*) ≤ 1 for 1 ≤ *i* ≤ *N* and 1 ≤ *k* ≤ 23, and so the factors *q*_*k*_(*i*) can be interpreted as probabilities. We point out, however, that any arbitrary choice of the constants *c*_*k*_, provided that they sum to *c*, will give the same transition probabilities in the product Markov chain and thus the results of the analysis do not depend on this choice.

### Incorporating whole genome duplication

It is possible to modify our model to allow for whole genome duplication [5]. To this end, consider an *N* × *N* matrix **G** with entries

$$G_{ij}=\{\begin{array}{cc} -p_{\text{gd}} & \text{if}\;i=j,\\ p_{\text{gd}}/2 & \text{if}\;2i=j,\\ 0 & \text{otherwise}, \end{array}$$

for 1 ≤ *i*, *j* ≤ *N*, where *p*_gd_ is a new parameter giving the probability that a random cell duplicates its genome but does not divide at a given generation.

To incorporate whole genome duplication, we use the matrices **M**_**gd**_ = **M** + **G** and **A**_**gd**_ = **DM**_**gd**_ instead of **M** and **A**, for the basic model and for the model with chromosome scores, respectively. With this modification, the corresponding Markov chains contain a transition from state *i* to 2*i* (or to the dead state if 2*i* > *N*) with probability *p*_gd_/2. Indeed, with probability *p*_gd_, a random cell duplicates its genome instead of producing two daughter cells, thus we can consider the transition probability to the “daughter” cell with duplicated genome to be *p*_gd_/2, while adding an additional transition to the dead state with probability *p*_gd_/2, corresponding to the other “daughter” cell that has not been created. It is possible to modify the matrix **G** to allow for the genome duplication probability *p*_gd_ to depend on the number of chromosome copies, by setting different values of *p*_gd_ for different rows of the matrix.

Since our model considers each of the 23 chromosomes independently, it cannot account for correlations between duplications in the different chromosomes (namely, the fact that all 23 chromosmes duplicate simultaneously). Nevertheless, by restricting to one chromosome at a time, the model gives the correct distribution of the number of copies over time, as well as the limiting distribution.

### Incorporating the effects of aneuploidy during early tumor growth

Aneuploidy and chromosomal instability are hallmarks of advanced solid tumors. However, during early stages of tumorigenesis, induction of aneuploidy has been shown to mitigate tumor growth [15, 16]. It was postulated that the negative effect of aneuploidy might be due to the various steps needed for tumor cells to become tolerant to chromosome copy number abnormalities. Loss of the tumor suppressor p53 has been shown to be a landmark event in the ability of mammalian cells to tolerate aneuploidy and complex karyotypes [17, 18]. In this section we attempt to model the process whereby key tumor suppressor proteins are inactivated either through mutational processes or copy number loss therefore enabling tolerance to chromosome missegregation.

To this end, we modify the Markov chain A by adding two additional states that model the early stage of the tumor, when deviation from a perfect diploid karyotype results in death due to the presence of active copies of a certain gene *X*. Recall that A contains *N* states corresponding to cells with *i* copies (for 1 ≤ *i* ≤ *N*) of a particular chromosome *k*, which we assume is the one containing gene *X*. To obtain the modified Markov chain, which we call $A_{X}$, the first additional state that we add to A corresponds to cells with two copies of chromosome *k*, both of which contain an active copy of gene *X*; we denote this state by *σ*. The second additional state corresponds to cells with two copies: one where gene *X* is active, and one where gene *X* is inactive due to mutation; we denote this state by *τ*.

Let *m*_*r*_ denote the mutation rate, which is the probability that, at a given generation, a given copy of chromosome *k* undergoes a mutation that inactivates gene *X*. The transition matrix of the modified Markov chain consists of the matrix **A** with two additional rows and columns, indexed *σ* and *τ*, and the following entries:

$$\begin{array}{c} \begin{array}{cc} A_{\sigma \sigma }=\left({\left(1-p\right)}^{46}-2m_{r}\right)q_{k}\left(2\right), & A_{\sigma \tau }=2m_{r}q_{k}\left(2\right),\\ A_{\tau \sigma }=0, & A_{\tau \tau }=\left({\left(1-p\right)}^{46}-m_{r}\right)q_{k}\left(2\right),\\ A_{\tau 1}=\frac{p}{2}q_{k}\left(2\right), & A_{\tau 2}=m_{r}q_{k}\left(2\right), \end{array} \end{array}$$

$$\begin{array}{c} \begin{array}{cc} A_{\sigma i}=A_{i\sigma }=A_{i\tau }=0 & \text{for}\;1\leq i\leq N,\\ A_{\tau i}=0 & \text{for}\;3\leq i\leq N. \end{array} \end{array}$$

Indeed, for a cell in state *σ*, the probability that either of the two active copies of gene *X* mutates (transitioning to state *τ*) is about 2*m*_*r*_. The entry *A*_*σσ*_ accounts for the fact that the cell dies if any of the 46 chromosome copies in the cell (2 for each of the 23 human chromosomes) missegregates. The probability of none of these copies missegregating is (1 − *p*)^46^. In the matrix, these probabilities are multiplied by the usual survival probability *q*_*k*_(2) of a cell with two copies of chromosome *k*. Similarly, for a cell in state *τ*, the probability that the active copy of gene *X* mutates (transitioning to state 2) is *m*_*r*_, and the probability that the active copy missegregates and a random daughter cell receives no active copies (transitioning to state 1) is *p*/2.

## Results

### Mathematical analysis of the basic model

Let (**M**^*g*^)_*i*,*j*_ be the entry in row *i* and column *j* of the *g*th power of **M**. In the one-chromosome version, this number is the proportion of cells after *g* generations that, starting with a founder cell that has *i* copies of a chromosome, have *j* copies of that chromosome. In particular, the sum of the entries of the *i*th row of **M**^*g*^, which we denote by *s*_*g*_(*i*), is the probability that the number of copies of the chromosome is between 1 and *N*.

When combining the 23 Markov chains to keep track of all chromosomes, the product $\prod_{k=1}^{23}s_{g}\left(n_{k}\right)$ is the surviving fraction after *g* generations when the founder cell has *n*_*k*_ copies of chromosome *k* for every *k*, as a fraction of 2^*g*^, which would be the number of cells after *g* generations if there were no deaths. Thus, $2^{g}\prod_{k=1}^{23}s_{g}\left(n_{k}\right)$ is the expected number of viable cells after *g* generations.

Restricting to viable cells, the *i*th row of **M**^*g*^ divided by *s*_*g*_(*i*) gives the probability distribution of the number of copies of a chromosome after *g* generations among viable cells, when the founder cell has *i* copies. More generally, if **v** is a probability vector that describes an initial distribution of the number of copies, then the vector **vM**^*g*^, divided by the sum of its entries, is the distribution among viable cells of the number of copies after *g* generations.

We are interested in the behavior of the Markov chain when the number of generations tends to infinity. Since the Markov chain M has an absorbing state, namely the one corresponding to dead cells, its stationary distribution is not very interesting: in the long run, the probability that a random branch ends at a dead cell tends to one. Instead, we would like to know the distribution of the number of chromosome copies *among viable cells*. Mathematically, we can do this by conditioning on not being on the absorbing state, and finding the limiting conditional distribution on the non-absobring states.

The Markov chain M has the property of being irreducible on the non-absorbing states, meaning that it is possible to go from any state other than the absorbing one to any other state if we allow enough steps. Markov chains with this property have been studied in the probability literature, see e.g. [19]. It is known that when conditioning on the non-absorbing states, the limiting conditional distribution of the chain is its so-called *quasi-stationary* distribution, which is unique. In our case, this is the unique *ρ*-invariant distribution for **M**, where *ρ* is its Perron—Frobenius (i.e. largest) eigenvalue. In other words, this distribution is the vector $v\in R_{\geq 0}^{N}$ satisfying **vM** = *ρ***v** and $\sum_{i=1}^{N}v_{i}=1$. We summarize this result as a lemma, since it will be used later on.

**Lemma 1**. *Let* Q *be a Markov chain with one absorbing state and N non-absorbing states*, *on which the chain is irreducible*. *Let* **Q** *be the transition matrix restricted to the non-absorbing states*, *and let ρ be its largest eigenvalue*. *Then*, *the limiting distribution of* Q *conditional on the non-absorbing states is given by the vector* $v\in R_{\geq 0}^{N}$ *satisfying* **vQ** = *ρ***v** *and* $\sum_{i=1}^{N}v_{i}=1$.

In particular, it follows from Lemma 1 that the limiting distribution of M conditional on the non-absorbing states does not depend on the number of chromosome copies of the founder cell. Next we show that, surprisingly, it does not depend on the missegregation rate *p* either. It will be convenient to write **M** as **M** = **I** + *p***J**, where **I** is the identity matrix, and **J** is the matrix with entries

$$J_{ij}=\{\begin{array}{cc} -i & \text{if}\;i=j,\\ i/2 & \text{if}\;|i-j|=1,\\ 0 & \text{if}\;|i-j|\geq 2, \end{array}$$ (2)

for 1 ≤ *i*, *j* ≤ *N*.

**Theorem 2**. *Assuming p* ≠ 0, *the limiting distribution of the Markov chain* M *conditional on the non-absorbing states is independent of p*.

*Proof*. Let us check that for *p* ≠ 0, the left eigenvectors of **M** of **J** are equal. Indeed, if **v** is a left eigenvector of **J** with eigenvalue λ, then **vJ** = λ**v**, which implies that **vM** = **v** + *p***vJ** = (1 + *p*λ)**v**, that is, **v** is a left eigenvector of **M** with eigenvalue 1+ *p*λ. The converse holds by a very similar argument.

In particular, the left eigenvector whose entries are nonnegative and sum to one having largest eigenvalue is the same for **M** and for **J**, and so it does not depend on *p*. By Lemma 1, such an eigenvector for **M** is the limiting distribution of the Markov chain on non-absorbing states.

From now on, for simplicity, the limiting distribution of M conditional on the non-absorbing states will simply be called the *limiting distribution* of M. Even though this distribution does not depend on *p* by Theorem 2, we will see later that the mixing time does, in the sense that the convergence to the limit distribution is slower if *p* is small.

Our next goal is to describe the limiting distribution of M. The following straightforward result from linear algebra will be useful when determining the eigenvectors of **M**.

**Lemma 3**. *For each n* ≥ 0, *let* **A**_*n*_ *be the tridiagonal matrix*

$$\begin{array}{c} A_{n}=[\begin{array}{cccccc} a_{1,1} & a_{1,2} & 0 & \cdots & 0 & 0\\ a_{2,1} & a_{2,2} & a_{2,3} & 0 & \cdots & 0\\ 0 & a_{3,2} & a_{3,3} & a_{3,4} & 0 & 0\\ \vdots & \ddots & \ddots & \ddots & \ddots & \vdots \\ 0 & \cdots & 0 & a_{n-1,n-2} & a_{n-1,n-1} & a_{n-1,n}\\ 0 & 0 & \cdots & 0 & a_{n,n-1} & a_{n,n} \end{array}], \end{array}$$

*where the entries a*_*i*,*j*_ *do not depend on n*, *and let P*_*n*_(*x*) = det(*x***I** − **A**_*n*_) *be its characteristic polynomial*. *Then the following hold*:

I *P*_*n*_(*x*) *satisfies the recurrence*
  $$\begin{array}{c} P_{n}\left(x\right)=\left(x-a_{n,n}\right)P_{n-1}\left(x\right)-a_{n,n-1}a_{n-1,n}P_{n-2}\left(x\right) \end{array}$$
  *for* *n* ≥ 2, *with initial conditions P*_0_(*x*) = 1 *and P*_1_(*x*) = *x* − *a*_1,1_.
II *Assuming that a*_*j*,*j*−1_ ≠ 0 *for all j*, *the left eigenvectors of* **A**_*n*_ *with eigenvalue* λ *have the form* **v** = (*v*_1_, *v*_2_, …, *v*_*n*_), *where*
  $$v_{i}=\frac{b\;P_{i-1}\left(\lambda \right)}{\prod_{j=2}^{i}a_{j,j-1}}$$
  *for* 1 ≤ *i* ≤ *n*, *and b* ≠ 0 *is a constant*.

*Proof*. The recurrence for *P*_*n*_(*x*) can be obtained easily by expanding the determinant along the last row.

To prove part II, note that for 1 ≤ *i* < *n*, the *i*-th component of the vector equation **vA**_*n*_ = λ**v** is

$$a_{i-1,i}v_{i-1}+a_{i,i}v_{i}+a_{i+1,i}v_{i+1}=\lambda v_{i},$$

where we write **v** = (*v*_1_, …, *v*_*n*_), and we let *a*_0,1_ = 0. Solving for *v*_*i*+1_, we get

$$v_{i+1}=\frac{1}{a_{i+1,i}}(\left(\lambda -a_{i,i}\right)v_{i}-a_{i-1,i}v_{i-1}).$$

It now follows by induction and using the recurrence for *P*_*n*_(*x*) that

$$v_{i}=\frac{P_{i-1}\left(\lambda \right)\;v_{1}}{\prod_{j=2}^{i}a_{j,j-1}}.$$

Letting *b* = *v*_1_ we get the stated expression for **v**.

Let *P*_*N*_(*x*) be the characteristic polynomial of the matrix **J** defined in Eq (2). Applying Lemma 3, we see that it satisfies the recurrence

$$P_{n}\left(x\right)=\left(x+n\right)P_{n-1}\left(x\right)+\frac{n(n-1)}{4}P_{n-2}\left(x\right)$$ (3)

with initial conditions *P*_0_(*x*) = 1 and *P*_1_(*x*) = *x* + 1. For example, for *N* = 8, we get

$$P_{8}\left(x\right)=x^{8}+36x^{7}+504x^{6}+3528x^{5}+13230x^{4}+26460x^{3}+26460x^{2}+11340x+2835/2.$$

**Fig 1:**
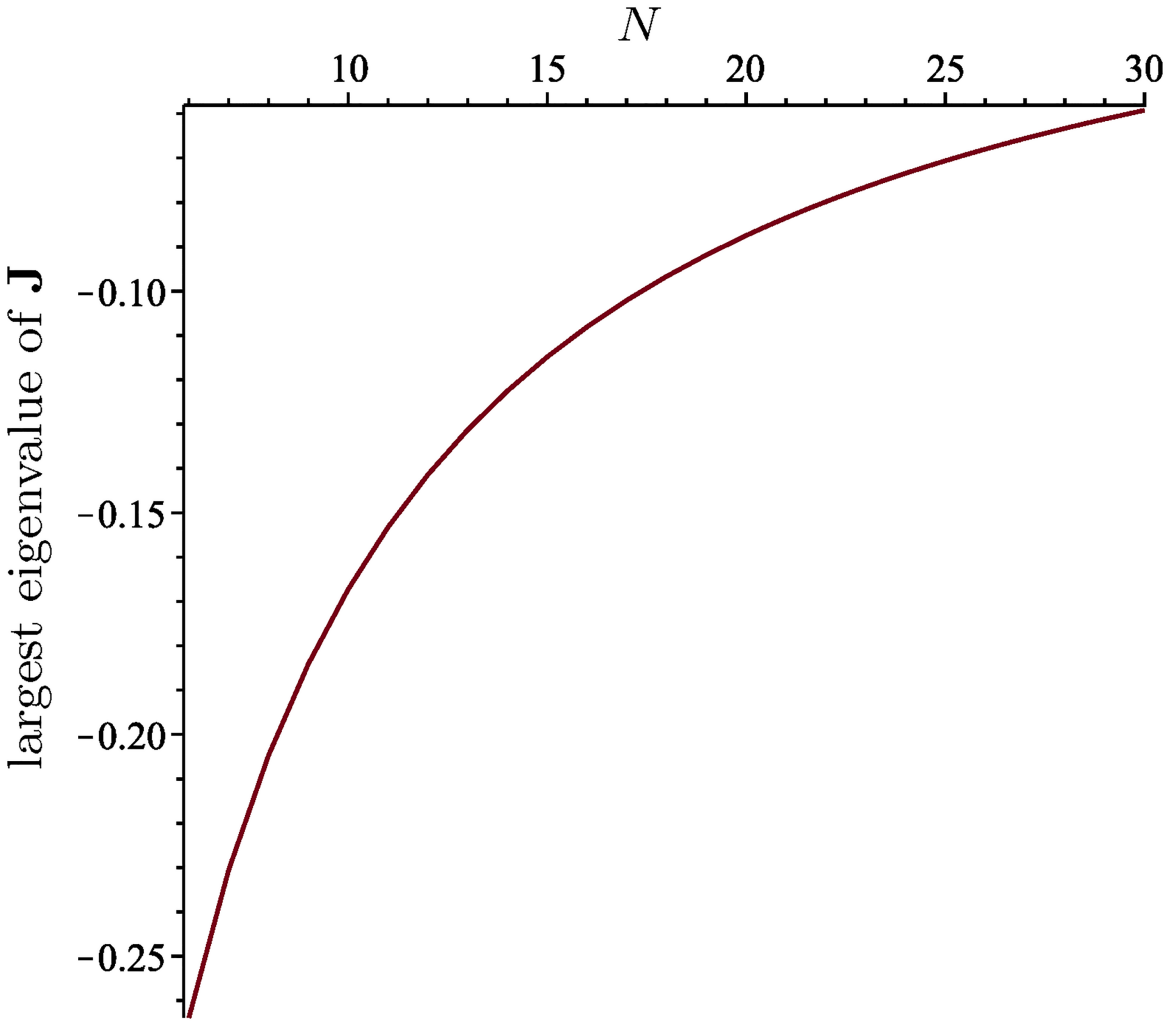
The largest eigenvalue of J as a function of *N*, for 6 ≤ *N* ≤ 30.

The largest eigenvalue of **J**, which is the largest root of *P*_*N*_(*x*), depends on *N*, as shown in Fig 1.

Using Lemmas 1 and 3, we can now describe the limiting distribution of M conditional on the non-absorbing states. The *i*-th component of **v** in the next theorem is the fraction of viable cells that have *i* copies of a given chromosome *k*, in the limit as the number of generations tends to infinity.

**Theorem 4**. *The limiting distribution of the Markov chain* M *conditional on the non-absorbing states is given by* $v=\frac{1}{\sum_{i=1}^{N}u_{i}}\left(u_{1},u_{2},\ldots ,u_{N}\right)$ *with*

$$u_{i}=\frac{2^{i-1}}{i!}P_{i-1}\left(\alpha \right),$$

*where the polynomials P*_*n*_(*x*) *satisfy recurrence* (3) *and α is the largest eigenvalue of* **J** *(equivalently, the largest root of P*_*N*_(*x*)).

*Proof*. By Lemma 1, the limiting distribution of M conditional on the non-absorbing states is given by the left eigenvector of **J** with largest eigenvalue *α*. The result now follows from Lemma 3, normalizing **v** so that its components sum to 1.

As shown in the proof of Theorem 2, if *α* is the largest eigenvalue of **J**, then 1 + *pα* is the largest eigenvalue of **M**. This eigenvalue determines the limiting growth rate of the tumor, which is the factor by which the number of viable cells multiplies at each generation assuming that karyotypes are distributed according to the limiting distribution. This growth rate is

$$2\;{\left(1+p\alpha \right)}^{23}.$$

**Fig 2:**
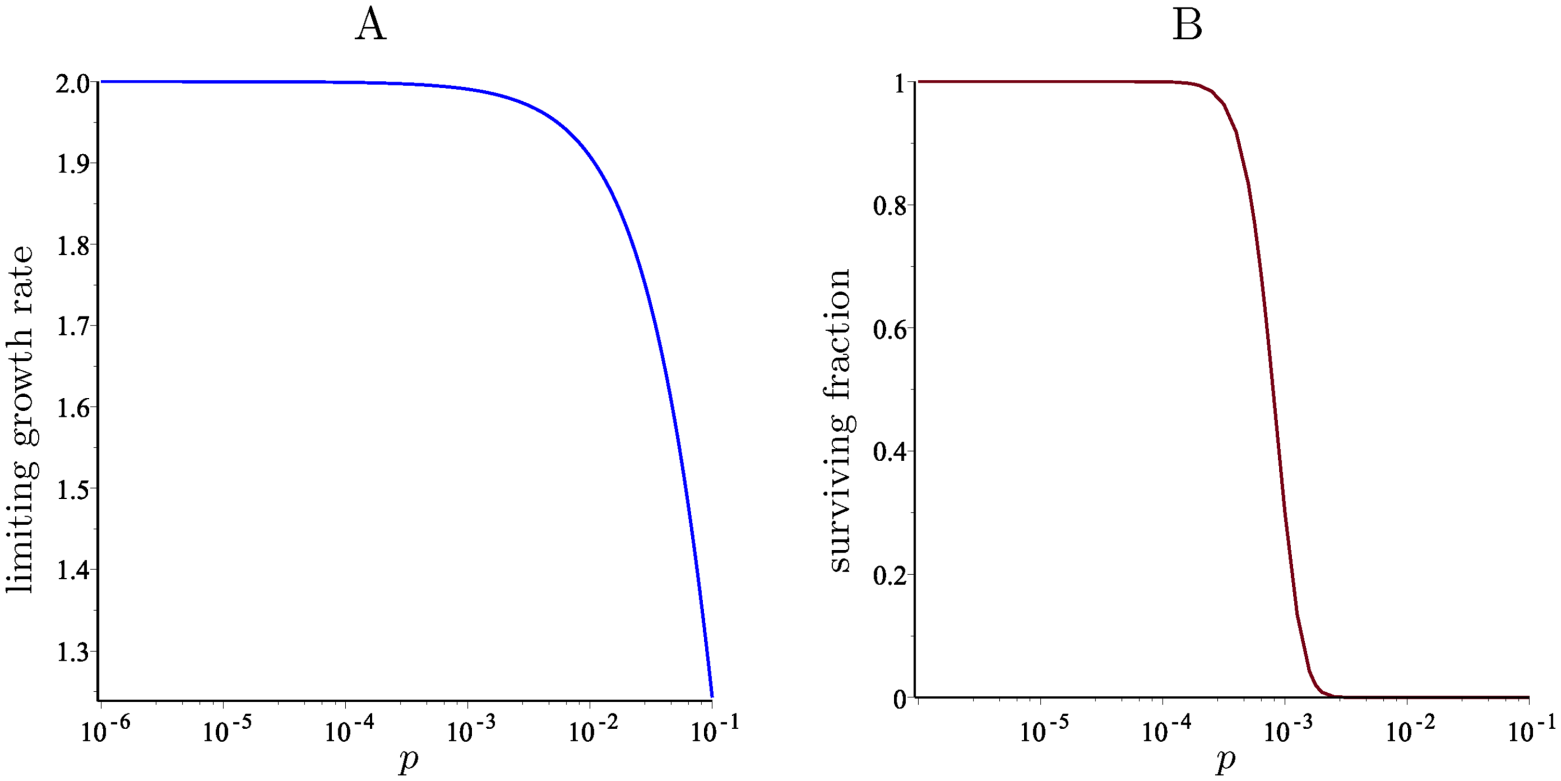
The limiting growth rate and surviving fraction in the basic model with *N* = 8. A: The limiting growth rate as a function of *p* (in a logarithmic scale). B: The fraction of cells that survive after 1000 generations, starting with a founder cell with 4 copies of each chromosome.

Fig 2A shows a graph of this function for *N* = 8 and varying *p*.

If we modified the model by allowing only a fraction *F* of the cells to survive at each generation, killing the remaining ones, then the reciprocal of the limiting growth rate, namely $\frac{1}{2\;{\left(1+p\alpha \right)}^{23}}$, would be the threshold such that for values of *F* below this threshold, the expected number of viable cells would tend to 0 as *g* → ∞, whereas for values of *F* above this threshold, the size of the colony would grow indefinitely.

Finally, Fig 2B shows the proportion of surviving cells, as a fraction of 2^*g*^, after *g* = 1000 generations for different values of *p*, starting from a cell with 4 copies of each chromosome. The fact that this fraction is close to 1 for very small values of *p* is another unrealistic prediction of the basic model, which will be addressed by the model with chromosome scores.

### Limiting distributions in the basic model

**Fig 3:**
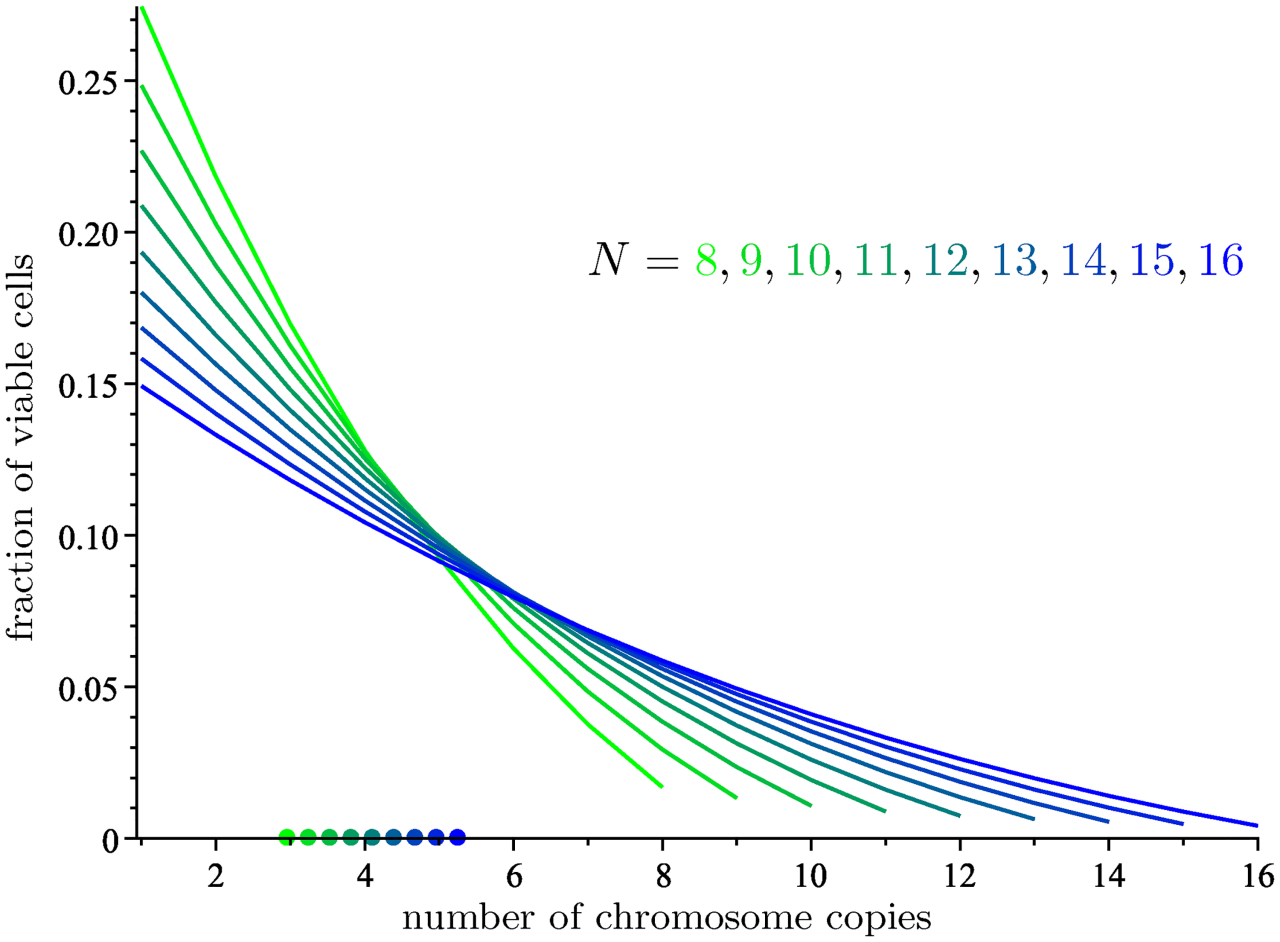
The limiting distribution of M on viable cells for 8 ≤ *N* ≤ 16. The average number of chromosome copies for each *N* is represented by a dot on the *x*-axis.

The limiting distribution described in Theorem 4 is computed in Table 1 for 6 ≤ *N* ≤ 10, along with its average, and graphed in Fig 3 for 8 ≤ *N* ≤ 16. For every *N*, the modal chromosomal number is 1, which agrees with the results of Gusev et al. [9], although it is not corroborated by experimental observations. In the next section we will describe a better model that will have more realistic outcomes. On the other hand, the average number of chromosome copies depends on *N*, and it is very close to 3 for *N* = 8.

**Table 1:** The limiting distribution of M on viable cells and average number of chromosome copies, for 6 ≤ *N* ≤ 10. The *i*th entry of each vector is the limiting fraction of viable cells with *i* copies of the chromosome.

*N*	limiting distribution of M conditional on non-absorbing states	average
6	(0.34691, 0.25538, 0.17996, 0.11850, 0.069129, 0.030127)	2.3980
7	(0.30638, 0.23576, 0.17598, 0.12582, 0.084111, 0.049851, 0.022079)	2.6832
8	(0.27432,0.21817,0.16968,0.12807,0.09262,0.06266,0.03760,0.01688)	2.9695
9	(0.24832, 0.20260, 0.16251, 0.12749, 0.09710, 0.07088, 0.04842, 0.02935, 0.01331)	3.2554
10	(0.22681, 0.18888, 0.15517, 0.12533, 0.09906, 0.07600, 0.05592, 0.03852, 0.02354, 0.01078)	3.5418

Even though Gusev et al. [9] guess from their figures that the chromosome copy numbers reach a “stable distribution” after a few hundred generations and that changes of *N* “do not affect the results of calculations,” we remark that the actual limiting distribution is heavily affected by the upper bound *N*. For example, while for *N* = 8 the limiting proportion of viable cells with one copy of the chromosome is about 0.27432 —which is close to the value observed in [9] with *p* = 0.1 after 200 generations—, for *N* = 200 this proportion is only 0.012984.

### Evolution of chromosome copy numbers over time in the basic model

**Fig 4:**
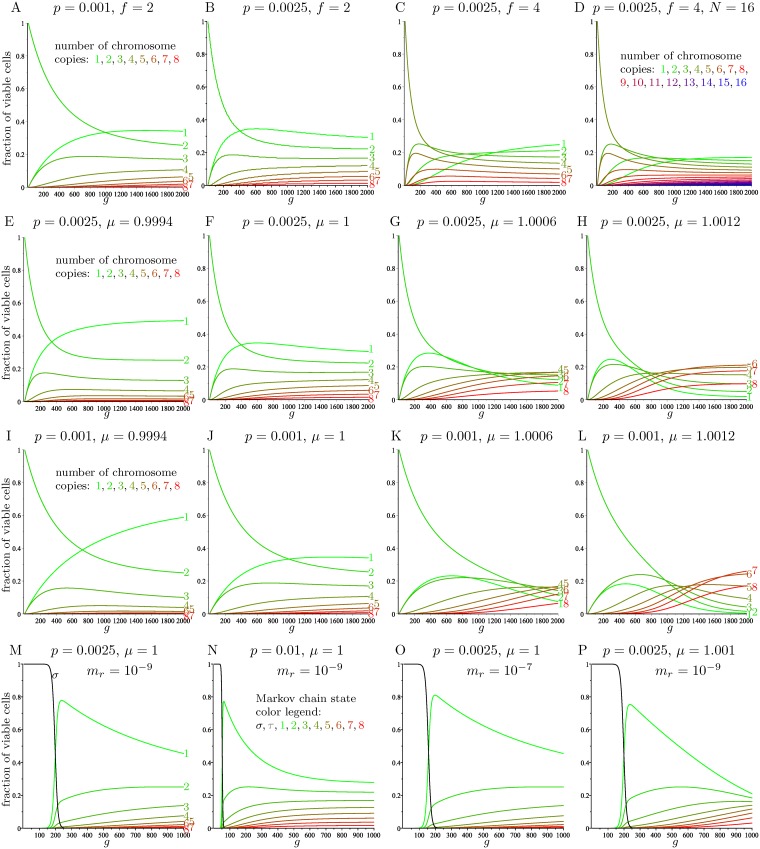
The evolution of the distribution of the number of chromosome copies for the various models. Each curve represents a given number of copies. A—D: Basic model (M) with *N* = 8 (except in D, which uses *N* = 16), over 2000 generations. E—L: Full model with chromosome scores (A) with *N* = 8 and a founder cell with *f* = 2 chromosome copies, over 2000 generations. M—P: Modified model incorporating the effects of aneuploidy during early tumor growth ($A_{X}$) with *N* = 8 and a founder cell with 2 active copies of gene *X*, over 1000 generations.

**Fig 5:**
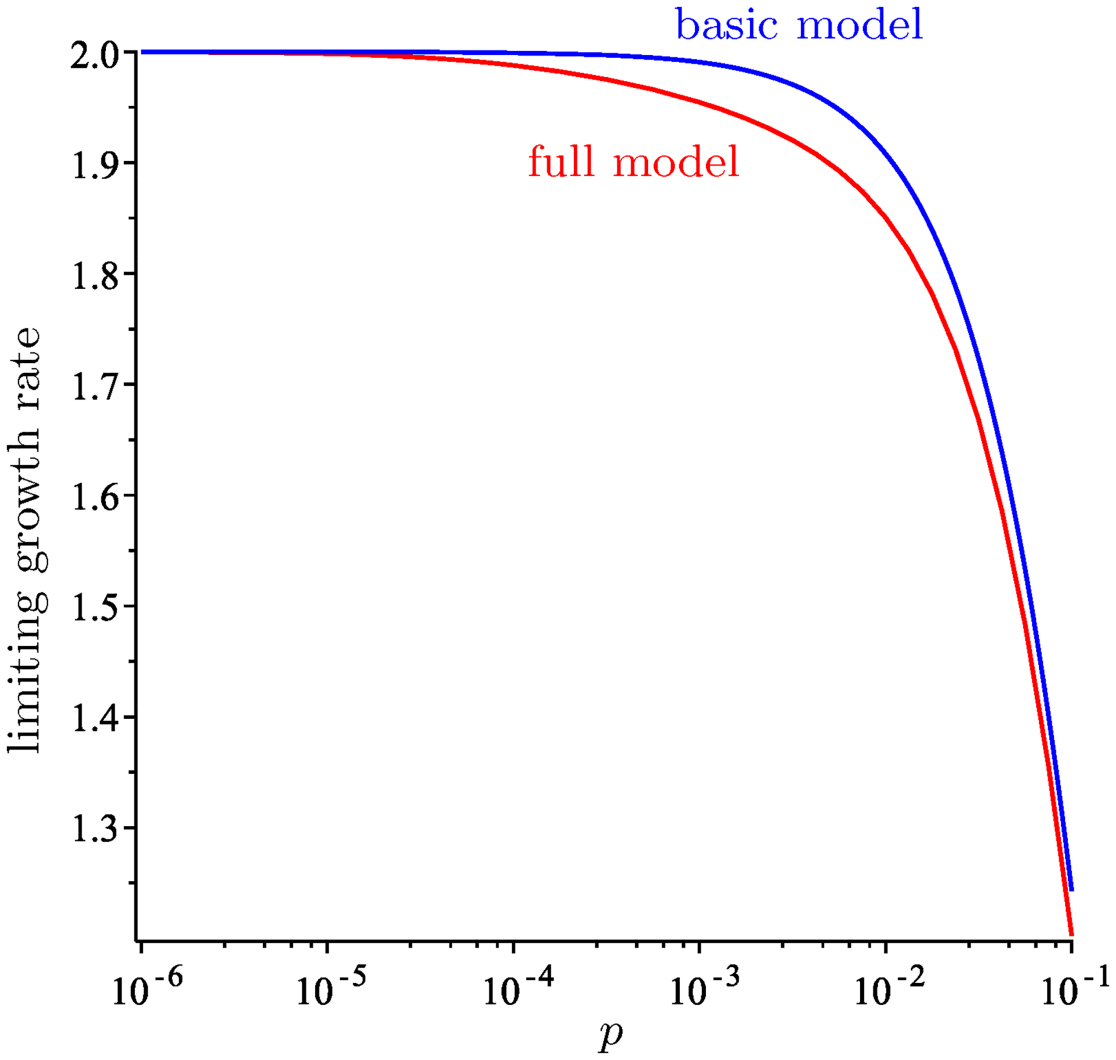
The limiting growth rate for the full model with *N* = 8 and the standard parameters. The limiting growth rate is graphed in red as a function of *p*. Fig 2A has been overlaid in blue for comparison.

Fig 4A–4D and S1 Fig show how the distribution of the number of copies of a chromosome evolves over time in the basic model, for different values of the missegregation rate *p*. The number of chromosome copies of the founder cell is denoted by *f*. S1 Fig replicates the data over 200 generations obtained by Gusev et al. [9, Figs 3A, 4A, 5A], showing that our simplification 3 does not noticeably affect the outcome for small values of *p*.

Fig 4A–4D shows data for 2000 generations. Note the similarity between Fig 4A and the center panel in S1 Fig. Indeed, for small values of *p*, increasing the number of generations by a factor of *s* has a similar effect to multiplying *p* by a factor of *s*. This is because (**I** + *p* **J**)^*s*^ ≈ **I** + *sp***J**. Fig 4D uses a different upper bound *N* = 16 on the allowed number of copies, and otherwise the same parameters as Fig 4C.

### Mathematical analysis of the full model

The *i*th row of the matrix **A**^*g*^, when normalized by dividing by the sum of the entries in the row, gives the distribution of the number of copies of chromosome *k* in viable cells after *g* generations, having started with a founder cell that has *i* copies of the chromosome. Note that before normalizing, the entries of **A**^*g*^ are affected by the choice of the constants *c*_*k*_. However, if we denote by $s_{g}^{\left(k\right)}\left(i\right)$ the sum of the entries of the *i*th row of **A**^*g*^, then the product $\prod_{k=1}^{23}s_{g}^{\left(k\right)}\left(n_{k}\right)$ is independent of this choice. The expression

$$2^{g}\prod_{k=1}^{23}s_{g}^{\left(k\right)}\left(n_{k}\right)$$

is the expected number of viable cells after *g* generations when the founder cell has *n*_*k*_ copies of chromosome *k* for every *k*.

As in the model without scores, the Markov chain A satisfies the conditions in Lemma 1. Thus, its quasi-stationary distribution, which is its limiting distribution conditional on the non-absorbing states, is given by the unique vector $v\in R_{\geq 0}^{N}$ satisfying **vA** = *ρ***v** and $\sum_{i=1}^{N}v_{i}=1$, where *ρ* is the largest eigenvalue of **A**. We call this the *limiting distribution* of A for simplicity, and we note that it does not depend on the number of chromosome copies of the founder cell.

However, the analogue of Theorem 2 no longer holds for A: its limiting distribution depends on *p*. As expected, it also depends on *μ* (equivalently, on the chromosome score), but not on the constant *c*_*k*_. Indeed, varying $C=e^{c_{k}/23}$ only changes **A** by a constant factor, which does not affect its eigenvectors. Another consequence is that while the number of viable cells in the colony after *g* generations depends on the parameter *c*, the limiting distribution of karyotypes among viable cells does not.

**Theorem 5**. *The limiting distribution of the Markov chain* A *conditional on the non-absorbing states is given by* $v=\frac{1}{\sum_{i=1}^{23}u_{i}}\left(u_{1},u_{2},\ldots ,u_{N}\right)$ *with*

$$u_{i}=\frac{2^{i-1}}{i!p^{i-1}\mu^{(i^{2}+i-2)/2}}P_{i-1}\left(\alpha \right),$$

*where the P*_*n*_(*x*) *satisfy the recurrence*

$$P_{n}\left(x\right)=\left(x-\mu^{n}\left(1-np\right)\right)P_{n-1}\left(x\right)-\mu^{2n-1}p^{2}\frac{n(n-1)}{4}P_{n-2}\left(x\right)$$

*with initial conditions P*_0_(*x*) = 1, *P*_1_(*x*) = *x* − *μ*(1 − *p*), *and α is the largest eigenvalue of* **A** *(i.e., the largest root of P*_*N*_(*x*)).

*Proof*. By Lemma 1, the limiting distribution of A conditional on the non-absorbing states is given by the left eigenvector of **A** with largest eigenvalue *α*. Since this eigenvector does not depend on the constant factor *C*, we can assume that *C* = 1, and so *q*_*k*_(*i*) = *μ*^*i*^. The entries of **A** are then

$$A_{ij}=\{\begin{array}{cc} \left(1-ip\right)\;\mu^{i} & \text{if}\;i=j,\\ ip\;\mu^{i}/2 & \text{if}\;|i-j|=1,\\ 0 & \text{if}\;|i-j|\geq 2. \end{array}$$

Applying Lemma 3 to **A**, it follows that its characteristic polynomial *P*_*N*_(*x*) satisfies the recurrence in the statement, and that its left eigenvector with eigenvalue *α*, normalized so that its components sum to 1, is **v**.

If *α*_*k*_ is the largest eigenvalue of **A**^(*k*)^, then the limiting growth rate of the tumor is

$$2\;\prod_{k=1}^{23}\alpha_{k}.$$ (4)

Its value depends on *p*, on the parameters *c*, *d*, and also on the scores of the 23 chromosomes.

The estimated values for these parameters that we will use in our figures are

c=-0.036132164andd=0.00039047. (5)

This value of *d* was found in [4] using experimental data. On the other hand, our value of *c* differs slightly from the value in [4] in order to ensure that *Q*_surv_ ≤ 1 for all valid karyotypes. Experimental values for the chromosome scores *s*_*k*_ were originally found in [8], and used in [4]. These values are given in Table 2, together with the corresponding values of $\mu =e^{ds_{k}}$.

**Table 2:** The values of the chromosome scores determined experimentally in [8], and the corresponding values of *μ*.

*k*	*s*_*k*_	*μ*
1	-0.143640496	0.999943914
2	0.638322635	1.000249277
3	0.597508197	1.000233336
4	0.106407616	1.000041550
5	-0.785208831	0.999693447
6	-0.664148445	0.999740704
7	3.039521587	1.001187547
8	1.650903175	1.000644836
9	0.765873656	1.000299095
10	-1.23443224	0.999518107
11	0.210103365	1.000082042
12	1.720482377	1.000672022
13	-1.207617162	0.999528573
14	-0.712581034	0.999721797
15	-0.751608856	0.999706562
16	-1.277797927	0.999501183
17	-0.784673321	0.999693656
18	-1.428496154	0.999442371
19	0.809097907	1.000315978
20	1.780741874	1.000695568
21	1.568732394	1.000612731
22	-1.576297101	0.999384693
23	0	1

Fig 5 shows a graph of the growth rate in Eq (4) as a function of *p*, with the values of *c*, *d* from Eq (5) and the chromosome scores from Table 2 (we call these the *standard parameters*). If we were to multiply *Q*_surv_ by a factor *F* to reduce the survival rate for all cells, then the reciprocal of expression (4) is the threshold for *F* that determines whether the expected number of cells will tend to zero or to infinity as *g* → ∞.

### Limiting distributions in the full model

The value of the parameter $\mu =e^{ds_{k}}$ in human chromosomes, using the estimates for chromosome scores from [8] and for *d* from [4], is roughly between 0.9994 and 1.0012 (see Table 2). We will use this range for *μ* in our computations below.

**Fig 6:**
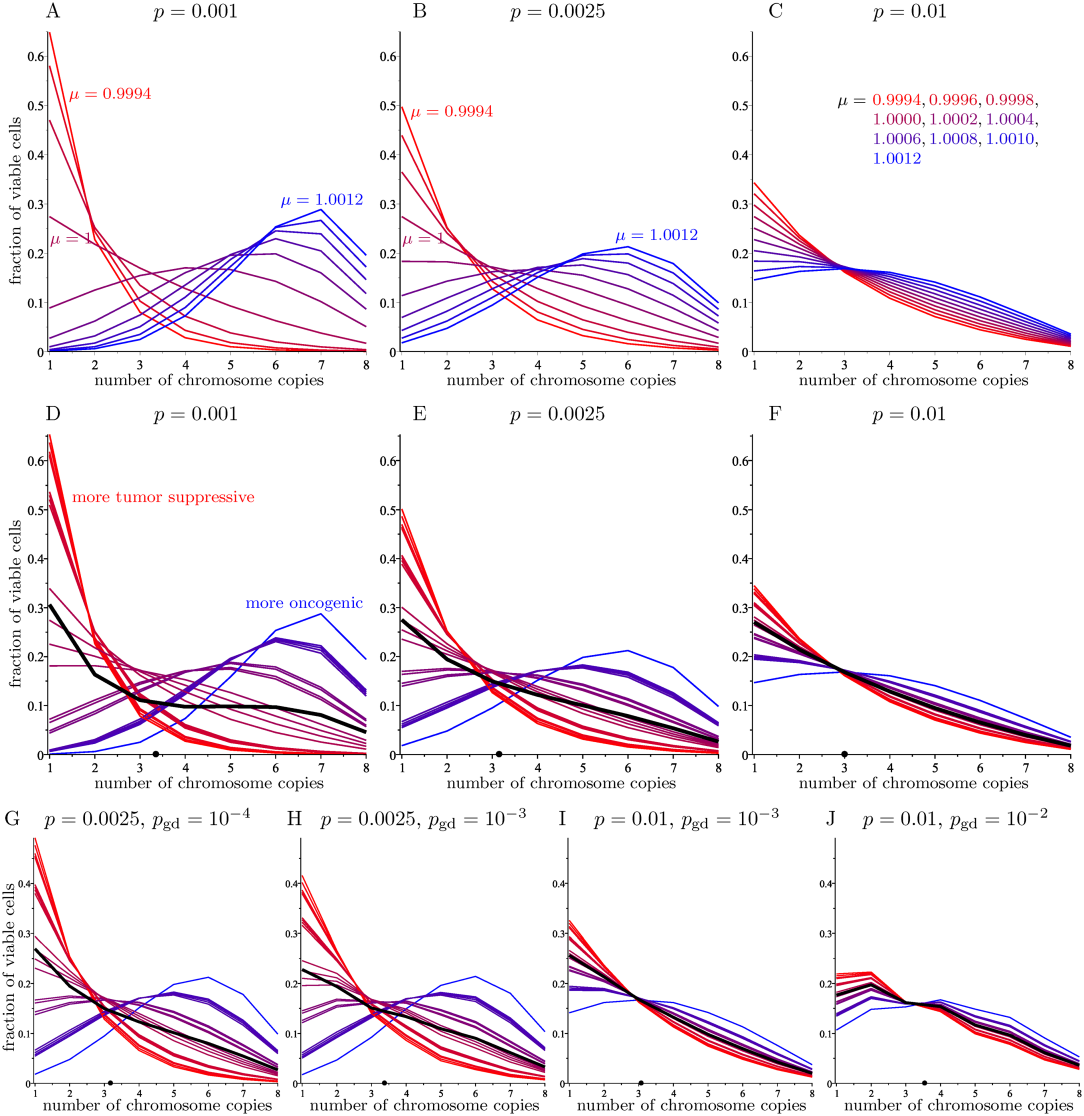
The limiting distribution on viable cells for the full model with *N* = 8. The horizontal axis indicates the number of copies of the chromosome, and the vertical axis measures the fraction of cells (among viable ones). A—C: Full model (A) for different values of *p*, and *μ* ranging in the interval [0.9994, 1.0012]. D—F: Full model (A) with the experimental values of *μ* corresponding to the 23 human chromosomes, for different values of *p*. The colors depict how oncogenic (blue) or tumor suppressive (red) each chromosome is. The average of the 23 limiting distributions is shown in black. The average number of chromosome copies in this average distribution is represented by a dot on the *x*-axis. G—J: Modified model with whole genome duplication for different values of *p* and *p*_gd_, together with the average of the 23 limiting distributions. The value *p*_gd_ = 0 corresponds to the full model depicted in panels D—F.

Fig 6A–6C shows the limiting distribution described in Theorem 5 for *N* = 8, three fixed values of *p*, and *μ* varying in the above range. Note that for *μ* = 1, which corresponds to a chromosome score of 0 (this is the score given to the sex chromosome), the limiting distribution is the same as in the basic model and it does not depend on *p*, since in this case **A** and **M** differ only by a constant factor.

**Fig 7:**
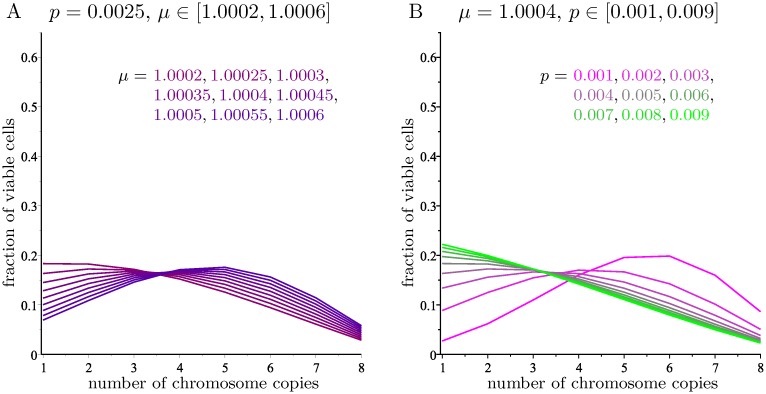
The limiting distribution of A with *N* = 8 for small variations of the parameters. A: For fixed *p* and varying *μ*. B: For fixed *μ* and varying *p*.

As expected, for higher chromosome scores, the limiting distribution favors higher numbers of copies. Smaller values of the missegregation rate *p* make the influence of the chromosome scores more noticeable, whereas larger values make the distribution closer to the one in Fig 3 for *N* = 8. It is interesting to observe that when the chromosome score is positive (equivalently, *μ* > 1), the modal number of copies soon becomes higher than one, and it gets larger as *μ* increases. This agrees with experimental observations and addresses the main shortcoming of Gusev’s model [9]. Fig 7A shows that for *p* = 0.0025, small variations of *μ* in the interval [1.0002, 1.0006] cause the modal number of copies in the limiting distribution to take all values between 1 and 5. The average number of copies and the modal number for three values of *p* and several values of *μ* is given in Table 3.

**Table 3:** The average and the modal number of chromosome copies in the limiting distribution of A on viable cells, for *N* = 8 and varying *p* and *μ*.

	*p* = 0.001		*p* = 0.0025		*p* = 0.01	
*μ*	average	mode	average	mode	average	mode
0.9994	1.538	1	1.975	1	2.613	1
0.9995	1.614	1	2.072	1	2.667	1
0.9996	1.713	1	2.189	1	2.722	1
0.9997	1.855	1	2.334	1	2.781	1
0.9998	2.071	1	2.511	1	2.842	1
0.9999	2.417	1	2.723	1	2.904	1
1.0000	2.969	1	2.969	1	2.969	1
1.0001	3.666	2	3.242	1	3.036	1
1.0002	4.288	4	3.526	1	3.102	1
1.0003	4.757	5	3.800	3	3.171	1
1.0004	5.104	6	4.057	4	3.242	1
1.0005	5.367	6	4.289	4	3.313	1
1.0006	5.573	6	4.492	5	3.382	1
1.0007	5.742	6	4.673	5	3.452	1
1.0008	5.883	6	4.832	5	3.523	1
1.0009	6.002	7	4.974	5	3.592	2
1.0010	6.105	7	5.101	6	3.661	2
1.0011	6.196	7	5.215	6	3.731	3
1.0012	6.277	7	5.316	6	3.795	3

Fig 6D–6F shows the limiting distribution for the experimental values of *μ* for each of the 23 human chromosomes (Table 2), for *N* = 8 and different values of *p*, as well as the average of these limiting distributions. The average number of chromosome copies in the limit is 3.3591 for *p* = 0.001, 3.1618 for *p* = 0.0025, and 3.0107 for *p* = 0.01. A graph of this dependence on *p* appears in S3A Fig.

We point out that, even though the basic model without chromosome scores also yielded an average number of chromosome copies near 3 for *N* = 8 (see Table 1), the shape of the limiting distribution in the basic model was unrealistic, with the modal number of copies always being 1.

The effect of changing the missegregation rate for a fixed chromosome score is shown in Fig 7B, which gives the limiting distributions obtained by fixing *μ* = 1.0004 (corresponding to a score of *s*_*k*_ = 1.0242) and letting *p* range from 0.001 to 0.009.

Next we analyze how these limiting distributions are affected by whole genome duplication. Considering the Markov chain with transition matrix **A**_**gd**_, Fig 6G–6J shows the limiting distribution of chromosome copy numbers for each of the 23 human chromosomes, for *N* = 8 and different values of both *p* and the genome duplication rate *p*_gd_. Comparing these results to those in Fig 6D–6F, which correspond to the case *p*_gd_ = 0 (i.e., no genome duplication), we see that, for rates of *p*_gd_ below 10^−4^, the outcomes are very similar to those of the model without whole genome duplication. On the other hand, larger values of *p*_gd_ skew the limiting distribution towards higher copy numbers, with this tendency being more noticeable when the missegregation rate *p* is low.

It is shown in [20] that certain karyotypes promote cytokinesis failure and thus genome duplication. In particular, it is suggested that cells with 3 or more copies of chromosome 13 have a higher genome duplication rate. This phenomenon can be incorporated in our model by using different values of *p*_gd_ in different rows of the matrix **G**. For example, making the value of *p*_gd_ increase by a factor of 10 when the number of copies of chromosome 13 is at least 3, the limiting distribution of the number of copies of chromosome 13 is shown in S4 Fig for different values of the parameters. We see that copy numbers 3 and above become more infrequent in this modified version, compared to the limiting distributions obtained when *p*_gd_ is independent of karyotype. Unfortunately, when *p*_gd_ is dependent on the number of copies of chromosome 13, our model cannot keep track of the distributions of other chromosomes.

### Evolution of chromosome copy numbers over time in the full model

As discussed above, the normalized rows of the powers of **A** describe the evolution over time of the distribution of the number of copies of a chromosome. This evolution is depicted in Fig 4E–4L for missegregation rates *p* = 0.0025 and *p* = 0.001, a founder cell with 2 copies of the chromosome, and different values of *μ*.

The number of generations that it takes for the distribution of chromosome copies to be close to the limiting distribution is determined by the mixing time of the Markov chain. This mixing time is roughly proportional to ${\left(1-\tilde{\rho }/\rho \right)}^{-1}$, where *ρ* and $\tilde{\rho }$ are the largest and the second largest eigenvalues of **A**, respectively. S2B Fig plots this quantity as a function of *p* for different values *μ*. Whereas the mixing time decreases for larger *p*, as expected, the dependence on *μ* is more subtle: values of *μ* further from 1 (in either direction) result in smaller mixing times. In the case *μ* = 1, which corresponds to the basic model with no chromosome scores, we have *ρ* = 1 + *pα* and $\tilde{\rho }=1+p\tilde{\alpha }$, where *α* and $\tilde{\alpha }$ are the two largest eigenvalues of **J**. The quantity ${\left(1-\tilde{\rho }/\rho \right)}^{-1}$ is plotted in S2A Fig for different values of *N*.

**Fig 8:**
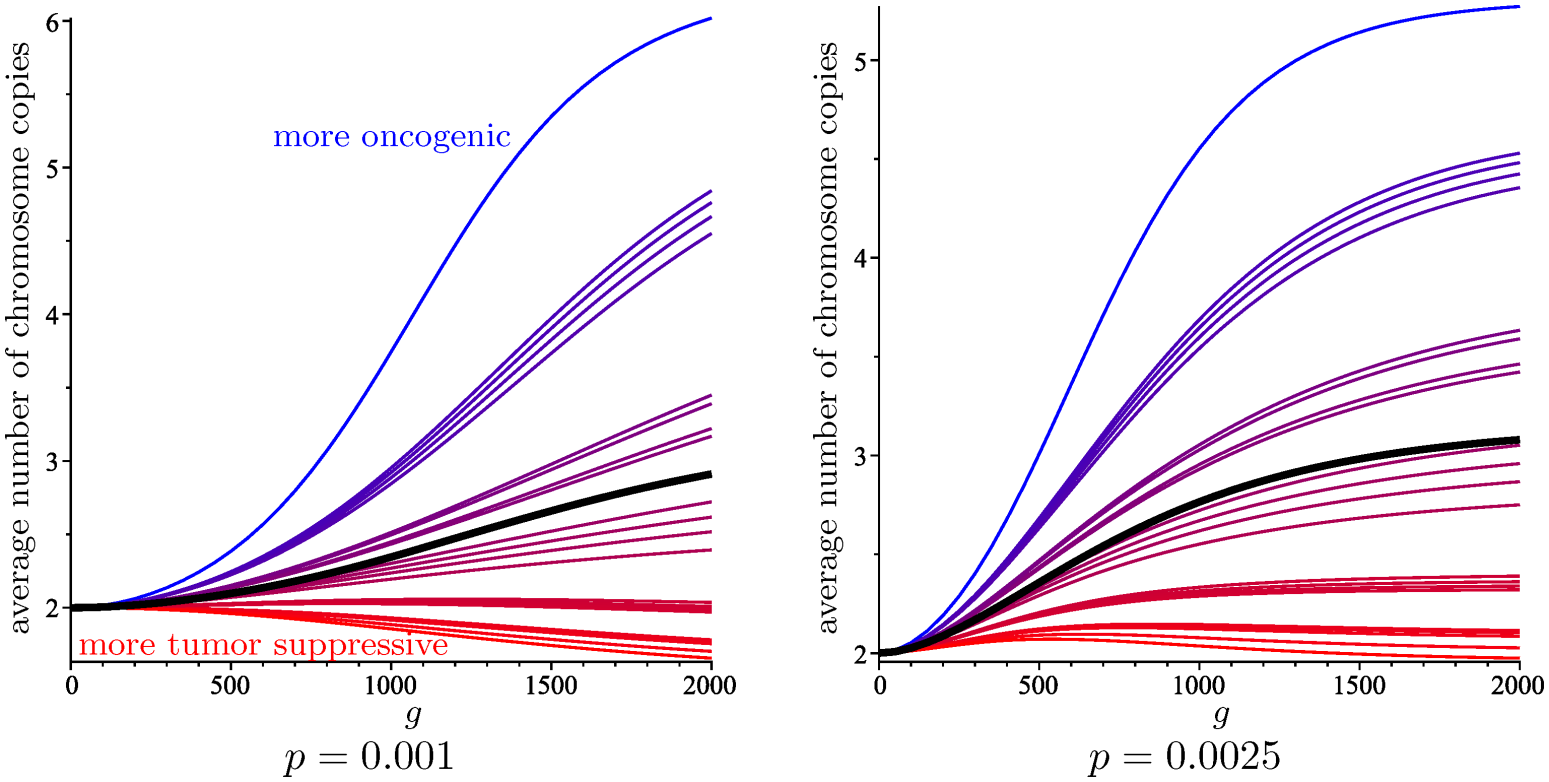
The evolution over 2000 generations of the average number of copies of the 23 human chromosomes, for *N* = 8 and two values of *p*. For each chromosome, the color of the curve depicts how oncogenic (blue) or tumor suppressive (red) it is. The average of the 23 averages is shown in black.

Fig 8 shows the evolution of the average number of copies of each of the 23 human chromosomes (with the scores from Table 2), as well as the total average number of copies for a random cell, with missegregation rates *p* = 0.001 and *p* = 0.0025, starting with a founder cell with 2 copies of each chromosome.

If we instead use the modified Markov chain $A_{X}$ that incorporates the effects of aneuploidy in early tumor growth, the evolution over time of the distribution of chromosome copy numbers is shown in Fig 4M–4P for different values of the parameters *p*, *m*_*r*_ and *μ*, when starting with a founder diploid cell with two active copies of gene *X*. These plots show that there is a sudden transition from the stage when most cells contain active copies of gene *X* (that is, states *σ* and *τ* in $A_{X}$), to the stage when most cells contain no active copies of gene *X* (that is, states 1, 2, …, 8). The value of *g* when this transition happens, which we call *time to inactivation*, is plotted in S5A Fig as a function of *p* and *m*_*r*_. We see that the time to inactivation is larger when *p* and *m*_*r*_ are small. S5B Fig displays the fraction of surviving cells (as a fraction of 2^*g*^) over time, showing that the growth rate of the colony sharply increases when inactivation takes place.

### Surviving fraction and heterogeneity in the full model

**Fig 9:**
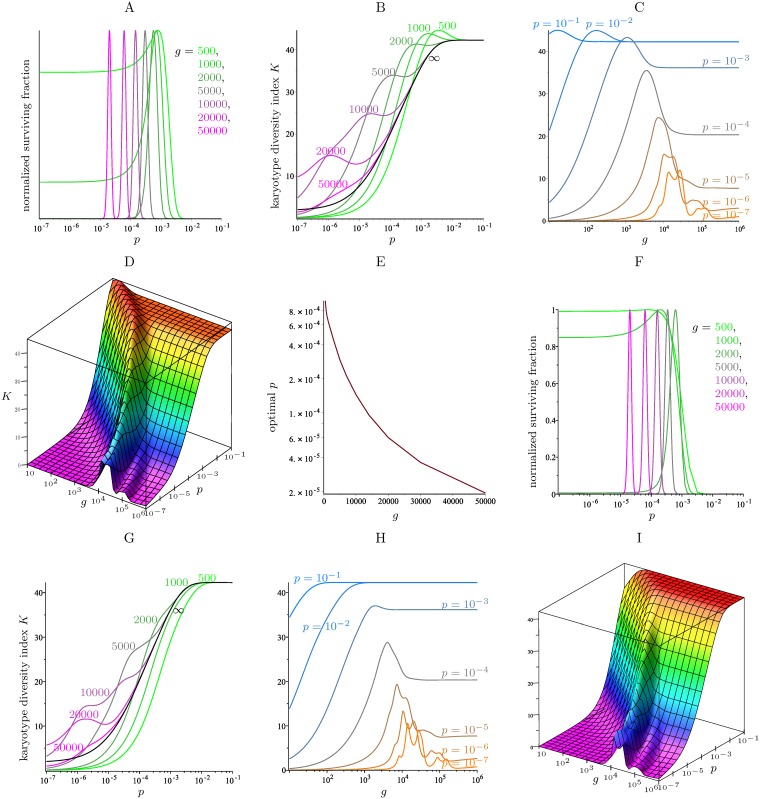
Surviving fraction and karyotype diversity index *K*, for the full model with *N* = 8 and the standard parameters. Starting with a founder cell with 4 copies (A—E) or 2 copies (F—I). A, F: Normalized fraction of cells that survive as a function of *p* (in a logarithmic scale) after *g* generations for seven values of *g*. B, G: Karyotype diversity index *K* for the same values of *p* and *g*. The black curve gives the karyotype diversity of the limiting distribution as a function of *p* (see this distribution in Fig 6D–6F for three values of *p*). As *g* → ∞, the other curves in the graph converge to the black curve. C, H: *K* as a function of *g* (in a logarithmic scale) for seven fixed values of the missegregation rate *p*. D, I: *K* as a function of *g* and *p* (both in a logarithmic scale), for 10 ≤ *g* ≤ 10^6^ and 10^−7^ ≤ *p* ≤ 10^−1^. E: The optimal value of *p* (in a logarithmic scale) that maximizes the surviving fraction times the karyotype diversity index *K* after *g* generations, as a function of *g*.

As we did in Fig 2B for the model without scores, we can compute the proportion of surviving cells, as a fraction of 2^*g*^, after *g* generations as a function of *p*. The corresponding graphs for different values of *g* are given in Fig 9A, starting from a cell with 4 copies of each chromosome and using the standard parameters (that is, *c* and *d* from Eq (5) and the chromosome scores from Table 2). The *y*-axis has been normalized for each graph so that the maximum surviving fraction occurs at the same height for each value of *g*. For *g* = 1000, a very similar figure appears in [4], where it was obtained by running lengthy computer simulations. The value of *p* that maximizes the fraction of cells that survive after *g* generations is just under 10^−3^ for *g* = 500 and *g* = 1000. This optimal value of *p* decreases slowly as the number of generation *g* increases. Interestingly, a large surviving fraction of cells is obtained only in a very narrow interval of values of the missegregation rate *p*, and this fact is more pronounced for large *g*.

Another important characteristic of the colony is its heterogeneity, which in [4] is measured as the Shannon diversity index of its cell scores. Here we propose another related measure of heterogeneity, based on the Shannon diversity of copy numbers of the different chromosomes. More precisely, if *a*_*k*,*j*_ denotes the fraction of viable cells in the colony with *j* copies of chromosome *k*, we define its *karyotype diversity index*

$$K=-\sum_{k=1}^{23}\sum_{j=1}^{N}a_{k,j}\text{ln}a_{k,j}.$$

In our model, the vector ${\left(a_{k,j}\right)}_{j=1}^{N}$ obtained after *g* generations starting with a founder cell with *i* copies of chromosome *k* can be easily computed by normalizing the *i*th row of **A**^*g*^. Fig 9B plots the karyotype diversity index *K* as a function of *p* for the same colonies as in Fig 9A, as well as the karyotype diversity index in the limiting distribution. After *g* = 500 and *g* = 1000 generations, the karyotype diversity is maximized when *p* is near 10^−3^, close to the value that maximizes the surviving fraction as well. For larger values of *g*, the curves in Fig 9B reach a local maximum that is not an absolute maximum, and this local maximum shifts to the left as *g* increases. The reason for this phenomenon is understood when considering *K* = *K*(*g*, *p*) as a function of two variables *g* and *p*. The graph of this function appears in Fig 9D. The cross sections for fixed *g* and varying *p* are the curves in Fig 9B, and the cross sections for fixed *p* and varying *g* are the curves in Fig 9C. For the latter curves, as *g* → ∞, the karyotype diversity index *K* converges to that of the limiting distribution for the given missegregation rate *p*. As the colony evolves towards this limiting karyotype distribution, it can attain values of *K* that are higher than the limiting value. For each fixed *p*, if we let *g*(*p*) be the value of *g* that maximizes *K*(*g*, *p*), then *g*(*p*) is a decreasing function of *p*. In other words, for smaller missegregation rates *p* it takes longer for the karyotype diversity to reach its maximum value. When fixing *g* and letting *p* vary, this effect translates into some of the curves in Fig 9B having a local maximum at the value of *p* such that *g* = *g*(*p*).

Fig 9D also illustrates that, in the region *g* ≤ 10^3^, the value of *K*(*g*, *p*) is nearly stable on the curves of the form *pg* = *constant*, attaining maximum values when this constant is close to 1. Interestingly, such high values of *K*(*g*, *p*) are only attained for missegregation rates *p* ≥ 10^−3^, after about *g* ≈ 1/*p* generations; in contrast, for lower missegregation rates, the karyotype diversity index does never reach such values, see Fig 9C.

Finally, we observe that, even though large missegregation rates *p* yield a high karyotype diversity index *K* (see Fig 9B), Fig 9A shows that the surviving fraction may be extremely low for such *p*. A measure of fitness is given by multiplying the surviving fraction from Fig 9A by the karyotype diversity index from Fig 9B. The value of *p* that maximizes this product is plotted in Fig 9E as a function of *g*.

If one starts with a founder cell with 2 copies of each chromosome, instead of 4 copies, the resulting data is shown in Fig 9F–9I, in analogy to Fig 9A–9D, respectively.

## Discussion

Herein, we have developed a Markov chain to directly analyze the long-term behavior of chromosome copy numbers in cancer cells whose viability and ability to evolve is shaped by numerical chromosomal instability—the frequent, yet understudied source of genomic instability in which cancer cells rapidly vary their karyotype through whole chromosome missegregation events during mitosis. Within the framework of this mathematical model, clonal fitness is defined by both the chromosomal distribution of oncogenes and tumor suppressor genes and the karyotype of single cells within the tumor population. Using this model, we directly obtain—without the need for lengthy computer simulations—the probability that a random cell after *g* generations has *i* copies of a specific chromosome, for any given *g*, *i* and an initial distribution of karyotypes. Further, we directly compute the expected size of a given clonal population after g generations when subject to selection pressures imparted by chromosomal instability. From a theoretical perspective, the main advantage of this Markov chain is that its stationary distribution can be used to determine the exact expected karyotype distribution of a population of cells after an infinite number of cell divisions. Conversely, exhaustive computational models can only approximately guess the behavior of single cell karyotypes in this limiting distribution. We therefore apply this model to precisely describe the limiting distributions of karyotypes in evolving clonal populations and discover the following:

1. The limiting distribution does not depend on the initial karyotype of founder cells or the probability of chromosome missegregation when the chromosomal distribution of oncogenes and tumor suppressor genes does not inform cell viability (i.e. the basic model). The limiting karyotype distribution of this basic model, is however, strongly affected by the upper bound placed on the maximum copy number of any specific chromosome that a viable cell can tolerate.
2. When cell viability is determined by the chromosome-specific distribution of tumor suppressor and oncogenes (i.e. the full model with chromosome scores), higher copy numbers of more oncogenic chromosomes are favored in the limiting distribution. This limiting distribution is still independent of the karyotype of founder cells. However, it depends now on the probability of chromosome missegregation.
3. Karyotype diversity within expanding clonal populations grows rapidly as a function of chromosome missegregation rates; however, very high missegregation rates are lethal to the cells because highly unstable clones are more likely to lose all copies of a given chromosome (or gain too many), which can lead to the complete loss of essential genes vital for cell survival. The selection imparted by the lethal effect of losing all copies of any given chromosome (nullisomy) generates an upper limit to karyotypic heterogeneity, which can be overcome only when given sufficient time for the population to evolve. This depends reciprocally on the number of cell divisions and the whole chromosome missegregation rate.
4. In an exponentially expanding clonal population, karyotypic heterogeneity is most exquisitely dependent on chromosome missegregation rates and its upward bounds are constrained by the risk for nullisomy. Whereas increased cell division number can lead to increased heterogeneity, at very low missegregation rates, even 10,000 generations of cell division fail to achieve maximal heterogeneity. This suggests that chromosome copy number heterogeneity observed in a given tumor is most likely influenced by chromosome missegregation rather than the age of the tumor.

The observation that maximal heterogeneity is most dependent on chromosome missegregation rates rather than the number of cell divisions has important implications toward our understanding of tumor evolution and therapy. It suggests that, at sufficiently high missegregation rates, heterogeneity can be readily obtained even during the early stages of tumorigenesis. Indeed, recent observations have demonstrated that pre-invasive lesions can achieve high levels of chromosome copy number abnormalities [21]. Furthermore, it was shown that pancreatic cancer evolution occurs in punctuated bursts of chromosomal alterations that generate significant heterogeneity over a short period of time thereby supporting metastatic progression [22]. This finding is also in line with observations showing that elevated chromosome missegregation rates in human tumors might be an important predictor of therapeutic resistance and existence of clonal heterogeneity irrespective of tumor stage [23].

### Comparison to other models in the literature

This Markov chain has several advantages over the computational models used by Laughney *et al*. [4] and in the previous papers [9, 10]. For example, it allows us to determine, without having to run lengthy computer simulations, the probability that a random cell after *g* generations has *i* of copies of a certain chromosome, for any given *g*, *i* and an initial distribution of karyotypes. It also yields the expected surviving fraction relative to an exponentially expanding population that does not undergo any cell death. From a theoretical point of view, the main advantage of the Markov chain is that its stationary distribution determines the exact expected distribution of copies of each chromosome as *g* tends to infinity. Note that the computational model can only make approximate guesses of the behavior in the limit. In this paper we compute the stationary distribution of the Markov chain, thereby obtaining a precise description of the limiting distribution of karyotypes, which agrees with prior observations [4].

This basic model is similar to the one considered by Gusev, Kagansky and Dooley [9, 24], which makes basic assumptions about how cells divide and missegregation events take place. Their stochastic model is developed whereby short-term simulations are run. That model uses a *semianalytical* approach to estimate the long-term behavior of the chromosome copy numbers in cancer cells. For this purpose, and to overcome some of the computational constraints of running the simulations, the authors develop a transition probability model similar to our Markov chain, which they run for as many as 500 generations, using the data to guess that there is a stable distribution in the limit.

Let us point out the main differences between the transition probability model used by Gusev *et al*. [9] and our Markov chain. The first difference is our simplification 3 described in the Methods section, which neglects quadratic terms in *p*. This simplification, which does not noticeably affect the behavior of the random process for small values of *p* like the ones observed in experiments, allows us to give an accurate and simple mathematical description of the limit behavior of the Markov chain. Another difference is our simplification 2, which allows us to interpret the entries of our transition matrix as probabilities of a Markov chain, and therefore apply theoretical results about Markov chains such as Lemma 1. Finally, the model by Gusev *et al*. [9] does not impose a realistic upper bound on the number of copies of a chromosome that a viable cell can have, which further complicates the computations, although a variation that imposes an upper bound is considered as well.

Based on the figures obtained from their simulations, Gusev et al. [9] observe that after a large enough number of generations (and for large enough *p*), the fraction of viable cells with *i* copies of a chromosome seems to converge for each *i*, but they give no mathematical proof of this phenomenon. One consequence of the analysis of our Markov chain is that we provide a proof of its convergence, and determine exactly what the limit values are. We also prove that these values do not depend on the missegregation rate *p* (in contrast to the “weak dependence on *p*” observed in [9] after 500 generations), or on the karyotype of the initial cell (this is also mentioned with no proof in the Gusev *et al*. model), although they do depend on the upper bound *N* on the number of allowed copies in viable cells.

In trying to remediate the fact that their model predicts a long-term distribution where the most likely number of copies of a chromosome is 1, which seems to disagree with experiments, Gusev *et al*. [9, §4.5.2] propose an alternative model which allows only one missegregation per chromosome type, as in simplification 3 above. However, this alternative model is significantly different from ours in that they consider the probability that a cell missegregates to be independent on how many copies of the chromosome it has. In practice, in a cell with more copies of a chromosome, it is more likely that some copy missegregates [25].

We remark that the basic model in this section also suffers from the same problem: it has a limiting distribution where the most frequent number of copies of a chromosome is 1. However, once we incorporate chromosome scores in the full model, we will obtain different limiting distributions that match the experimentally observed ones.

Finally, a continuous time model based on the one from Gusev *et al*. [9, 24] was developed by Desper, Difilippantonio, Ried and Schäffer [10]. This model uses an exponential distribution for the time between cell divisions, and it allows to vary the cell division rates as a function of the number of copies of the chromosome. In this study, the authors consider the evolution of the average copy number, and obtain some analytic estimates for it.

### Potential uses for our Markov chain model

Predicting tumor behavior from single-cell data is critical to our ability to simulate complex processes such as therapeutic resistance. Significant effort has been devoted toward simulating mutational processes in cancer in an attempt to predict resistance to targeted therapies for example. However, these efforts have not incorporated numerical chromosomal instability, a major driver of therapeutic resistance. Our Markov chain can be integrated with other models to account for both mutational heterogeneity as well as chromosome copy number evolution. Integrated models that combine different modes of genomic instability would undoubtedly be better at predicting the process of therapeutic resistance. Such models would generate experimentally testable hypothesis in the laboratory and would be used as a guide to inform clinical management and the selection of anti-cancer therapies.

## Supporting information

S1 Fig The distribution of the number of chromosome copies in the basic model with *N* = 8 over 200 generations, for different values of *p* and *f*. (TIFF)

S2 FigThe value of ${\left(1-\tilde{\rho }/\rho \right)}^{-1}$, which is an estimate of the mixing time of the Markov chain, as a function of *p*.A: Basic model (M), for 8 ≤ *N* ≤ 16. B: Full model (A) with *N* = 8 and *μ* in the range [0.9994, 1.0012]. The curve for *μ* = 1, which has been truncated, coincides with the lowest curve in A.(TIFF)

S3 FigThe average number of chromosome copies, averaged over the 23 limiting distributions for experimentally computed human chromosome scores.A: In the full model (A), as a function of *p*. B: In the modified model with whole genome duplication, as a function of *p* and *p*_gd_.(TIFF)

S4 FigThe limiting distribution of copies of chromosome 13 in the modified model with whole genome duplication, with *p*_gd_ dependent on the number of copies.The dashed line shows the limiting distribution when the genome duplication rate is $p_{\text{gd}}^{\leq 2}$ or $p_{\text{gd}}^{\geq 3}$ depending on whether the number of copies of chromosome 13 is at most 2 or at least 3, respectively. The solid line shows the limiting distribution when the genome duplication rate *p*_gd_ is constant (these are the same curves given in Fig 6H–6J for chromosome 13).(TIFF)

S5 FigTime to inactivation and surviving fraction for the model incorporating the effects of aneuploidy during early tumor growth ($A_{X}$) with *N* = 8 and a founder cell with 2 active copies of gene *X*.A: Time to inactivation, i.e. the number of generations until the proportion of cells containing no active copies of gene *X* is more than half, as a function of *p* and *m*_*r*_. B: Surviving fraction over 500 generations.(TIFF)
