# Supplementary figures and images for: A Markov chain for numerical chromosomal instability in clonally expanding populations
Source: PLoS Comput Biol. 2018 Sep 11;14(9):e1006447. doi: 10.1371/journal.pcbi.1006447 (PMC6150543; doi:10.1371/journal.pcbi.1006447)

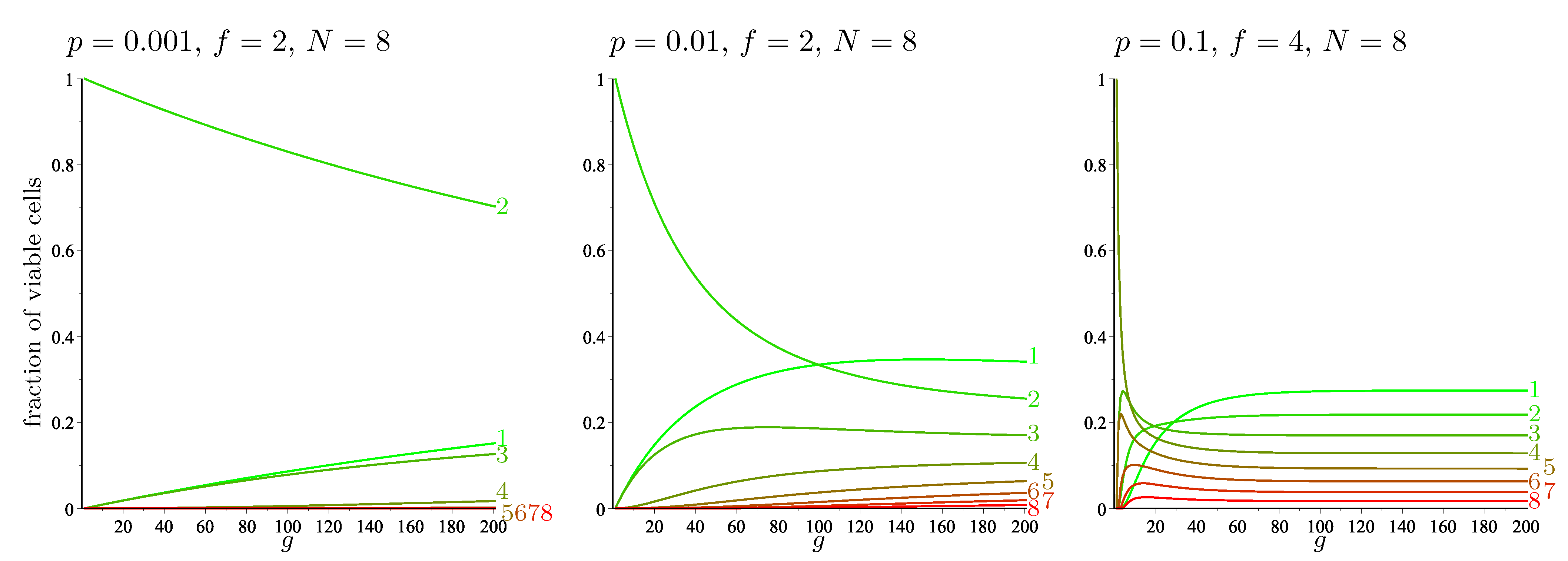

Supplement: S1 Fig — (TIFF) [file pcbi.1006447.s001.tiff]

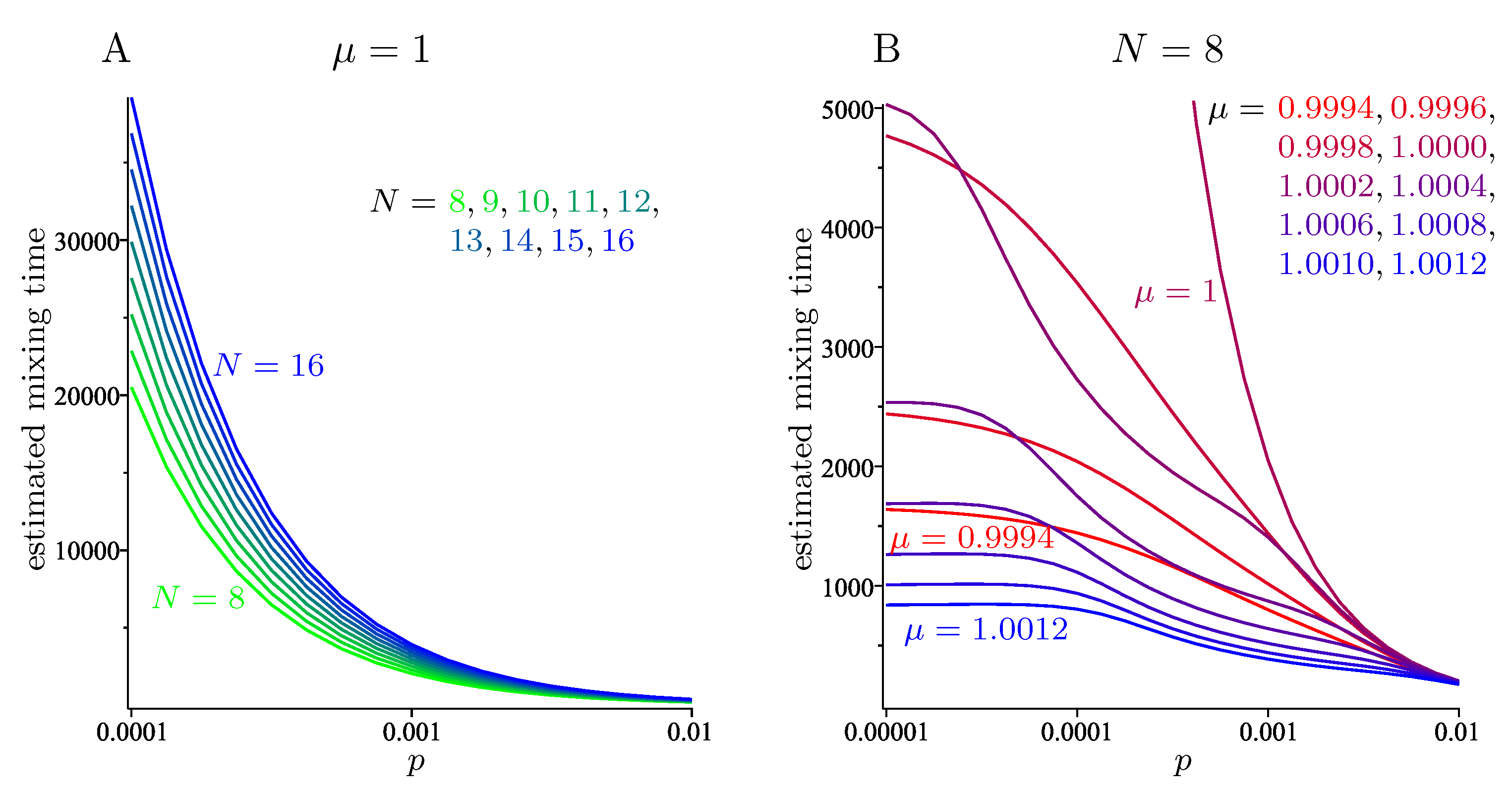

Supplement: S2 Fig — A: Basic model (M), for 8 ≤ N ≤ 16. B: Full model (A) with N = 8 and μ in the range [0.9994, 1.0012]. The curve for μ = 1, which has been truncated, coincides with the lowest curve in A. (TIFF) [file pcbi.1006447.s002.tiff]

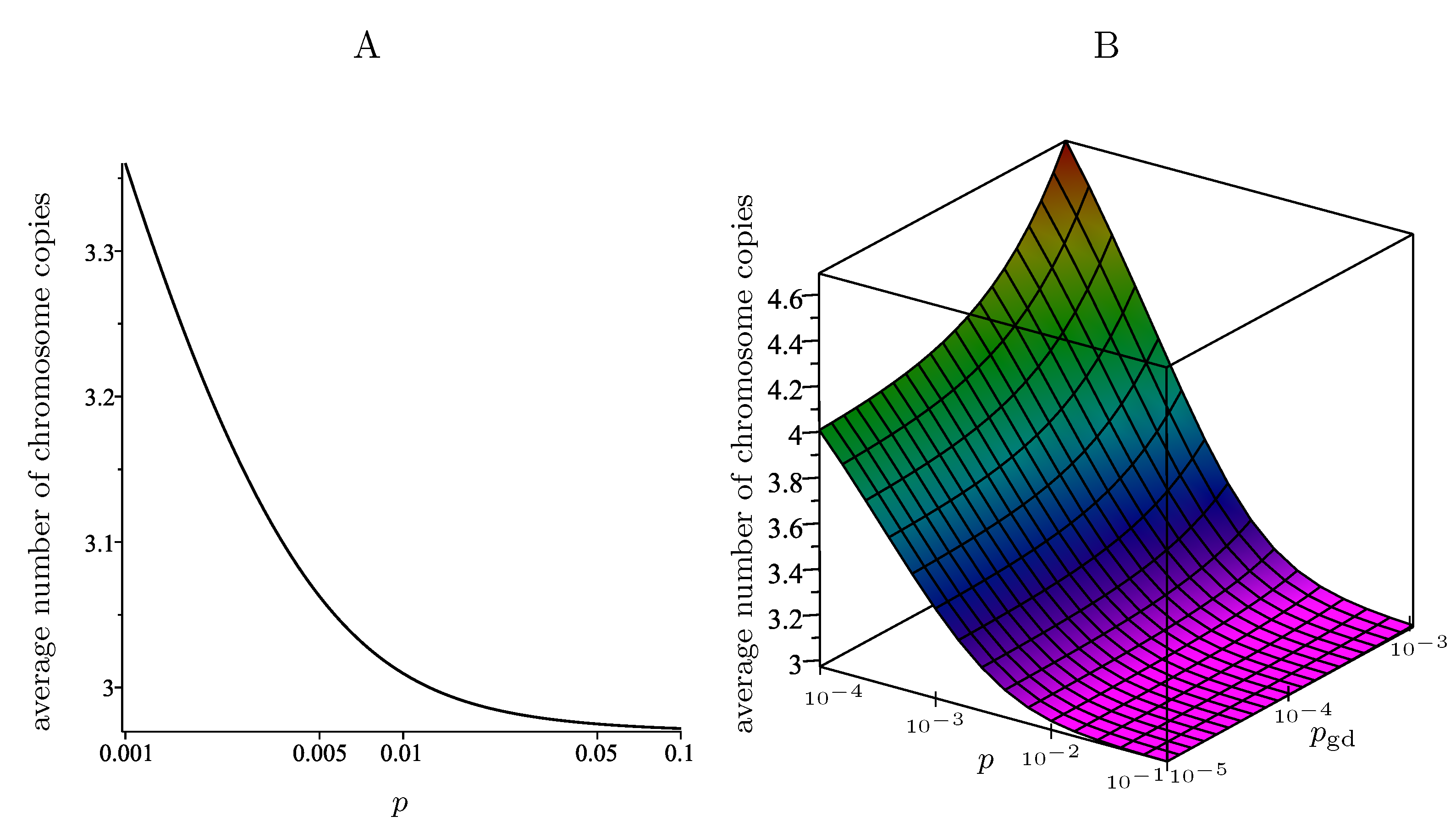

Supplement: S3 Fig — A: In the full model (A), as a function of p. B: In the modified model with whole genome duplication, as a function of p and pgd. (TIFF) [file pcbi.1006447.s003.tiff]

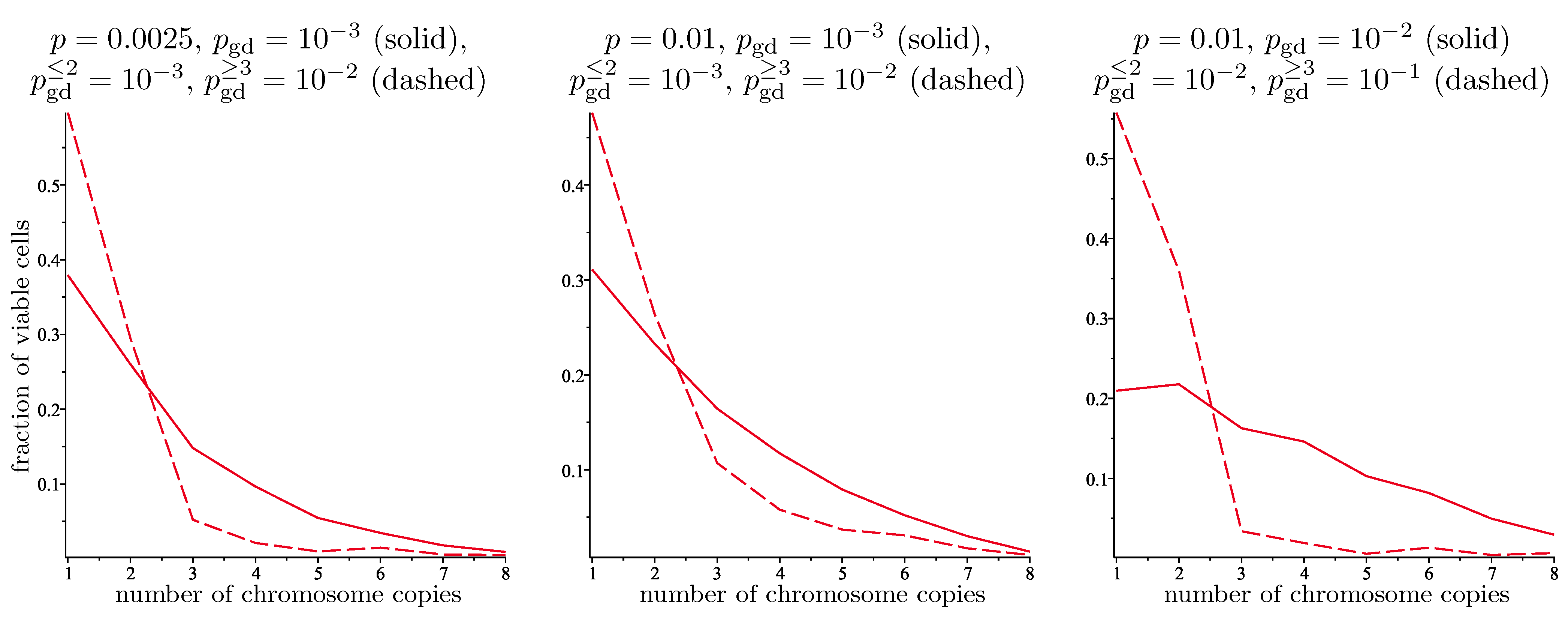

Supplement: S4 Fig — The dashed line shows the limiting distribution when the genome duplication rate is pgd≤2 or pgd≥3 depending on whether the number of copies of chromosome 13 is at most 2 or at least 3, respectively. The solid line shows the limiting distribution when the genome duplication rate pgd is constant (these are the same curves given in Fig 6H–6J for chromosome 13). (TIFF) [file pcbi.1006447.s004.tiff]

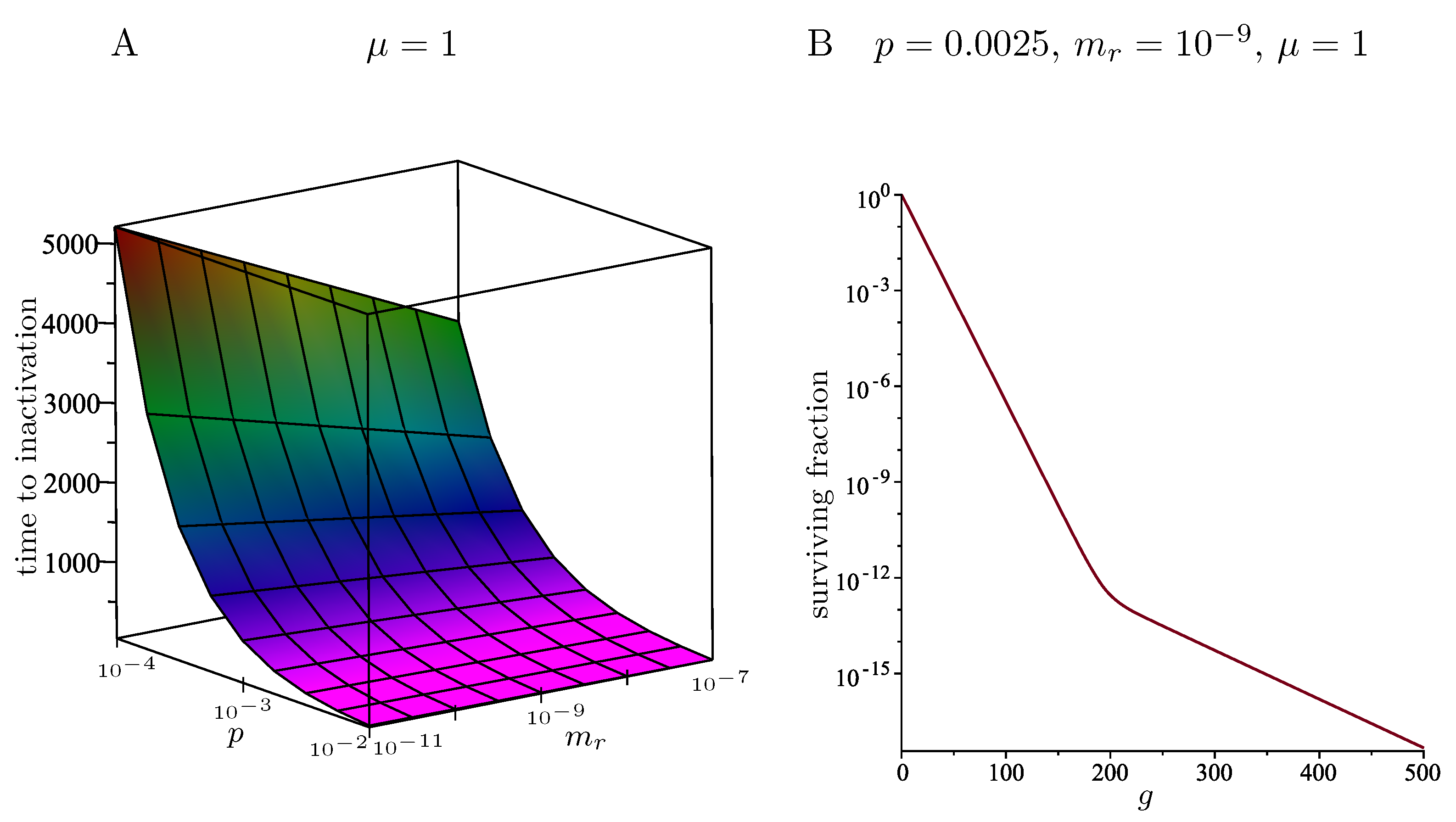

Supplement: S5 Fig — A: Time to inactivation, i.e. the number of generations until the proportion of cells containing no active copies of gene X is more than half, as a function of p and mr. B: Surviving fraction over 500 generations. (TIFF) [file pcbi.1006447.s005.tiff]
